# Optimizing Wheat Yield, Water, and Nitrogen Use Efficiency With Water and Nitrogen Inputs in China: A Synthesis and Life Cycle Assessment

**DOI:** 10.3389/fpls.2022.930484

**Published:** 2022-06-16

**Authors:** Zhou Li, Song Cui, Qingping Zhang, Gang Xu, Qisheng Feng, Chao Chen, Yuan Li

**Affiliations:** ^1^Key Laboratory of Animal Genetics, Breeding and Reproduction in the Plateau Mountainous Region, Ministry of Education, College of Animal Science, Guizhou University, Guiyang, China; ^2^School of Agriculture, Middle Tennessee State University, Murfreesboro, TN, United States; ^3^College of Agriculture and Forestry Science, Linyi University, Linyi, China; ^4^The State Key Laboratory of Grassland Agro-Ecosystems, College of Pastoral Agriculture Science and Technology, Lanzhou University, Lanzhou, China

**Keywords:** decision tree, irrigation, life cycle assessment, meta-regression, structural equation modeling (LISREL)

## Abstract

To meet the demand of the fast increasing population, enhancing the wheat (*Triticum aestivum* L.) yield and resource use efficiency by optimizing water and nitrogen (N) management can greatly improve agricultural sustainability and enhance regenerative farming in developing countries such as China. Based on 126 studies conducted in China between 1996 and 2018, using meta-analysis in combination with decision regression tree modeling and life cycle assessment (LCA), this study aimed to (1) quantify the effect of water and N input on wheat yield, water productivity (WP_*c*_), and N use efficiency (NUE_*f*_), and evaluate the subsequent environmental impact in different regions using LCA; and (2) evaluate, model, and rank the roles of environmental (e.g., soil nutrient status and climatic factors) and agronomic factors (e.g., water and N management practices) affecting wheat yield, WP_*c*_, and NUE_*f*_. The results showed that irrigation and N addition increased the average yield and WP_*c*_ by 40 and 15%, respectively, relative to control treatments with no irrigation or fertilizer application. The mean water saving potential (WSP) and N saving potential (NSP) in China were estimated at 11 and 10%, respectively. Soil nutrient status [e.g., initial soil phosphorus (P) and potassium (K)] and soil organic carbon content affected the wheat yield, WP_*c*_, and NUE_*f*_ more significantly than climatic factors [mean annual temperature (MAT)] or water and N management practices. The structural equation-based modeling indicated that initial soil nutrient condition impacted productivity and resource use efficiency more at the below optimal water and N levels than above. The risk-factor-based feature ranking indicated that site-specific environmental and soil condition was highly informative toward model construction but split input of N or water had less impact on yield and input use efficiency. LCA demonstrated that to further mitigate greenhouse gas emissions, water- or N-saving management should be promoted in China. Collectively, our research implies that long-term soil health and nutrient enhancement should be more beneficial for increasing yield and resource use efficiency in wheat production.

## Introduction

Wheat (*Triticum aestivum* L.) is a cool-season C_3_ cereal crop commonly produced worldwide. It is considered the second most important crop in developing countries followed by rice (*Oryza sativa* L.) (Narayanan, 2018). Comparable with other C_3_ cereal crops, the productivity and agronomic input use efficiency of wheat is largely affected by many environmental and managerial factors, such as growing season temperature, irrigation methods, and N fertilization rate (Fan et al., 2018). In addition, a lot of fossil fuel energy is used for producing agronomic inputs, such as chemical fertilizers, leading to severe environmental pollution, particularly, in those developing countries (Taki et al., 2018). Information abounds with individual field studies investigating the impacts of one or two factors on wheat productivity and physiology at a single site (Zwart and Bastiaanssen, 2004; Qin et al., 2015). However, round evaluation of numerous factors at the same time requires systematic data synthesis and modeling effort using data at a much larger spatial and temporal scale.

China has been the leading wheat-producing country in the world since 1991. According to FAOSTAT (2017), the total wheat production area in China ranked third globally in 2014 [24 million hectares (ha)], closely following the European Union (27 million ha) and India (31 million ha). The wheat yield in China averagely increased five times from 1949 (<1 t ha^–1^) to 2013 (5 t ha^–1^) (Huang et al., 2015). Despite the advancement of modern breeding efforts and technological advancement (e.g., irrigation technology and synthetic fertilizers), the production capacity of wheat in China struggles to meet the skyrocketing demand caused by fast population increase, urbanization, and diminishing land and natural resources (Du et al., 2020). In addition, intensive monoculture-based wheat cropping systems largely rely on agronomic inputs [e.g., water and nitrogen (N)], which have significantly challenged the overall ecological and economic sustainability while maintaining productivity on a system level (Conway and Toenniessen, 1999; Lollato et al., 2019; Sidhu et al., 2019). Thus, there is a growing demand for identifying key managerial and environmental drivers affecting wheat yield, water productivity (WP_*c*_), and N use efficiency (NUE_*f*_) with data collected across large spatial and temporal scales to enhance agronomic and environmental sustainability (Tilman et al., 2011; Li et al., 2019). The decision tree-based regression has been involved in agricultural research to identify key factors affecting agriculture production and could be of great use in future field experiments; e.g., decision tree analyses were used to find the key predictors for characterizing soybean seed yield from commonly collected precision agriculture across Wisconsin, United States (Smidt et al., 2016).

Small grain crops, such as wheat, are highly dependent on N fertilization, which consequentially resulted in greater crude protein concentration and nutritive value compared with C_4_ crops (Raun and Johnson, 1999; Gonzalez-Dugo et al., 2010). However, over-fertilization could result in low NUE_*f*_ and groundwater contamination under unfavorable environmental conditions (Tilman et al., 2011; Lollato et al., 2019; Sidhu et al., 2019). Ladha et al. (2016) reported that, on average, wheat production consumes 18% of global N fertilizer, with a 50-year average of 52 kg ha^–1^ year^–1^. Meanwhile, the average NUE_*f*_ across major cereals is lower than 33% (Raun and Johnson, 1999). The global average of wheat WP_*c*_ was 1.09 kg m^–3^, ranging from 0.6 to 1.7 kg m^–3^ (Zwart and Bastiaanssen, 2004). High yield variability suggests that wheat is mostly affected by genetic, environmental, or management differences. Thus, there exists enormous potential for improving WP_*c*_ and NUE_*f*_ through breeding and better management. Several studies have endorsed that the underlying interactive mechanisms impacting the WP_*c*_ and NUE_*f*_ of wheat would be complex (Zwart and Bastiaanssen, 2004; Qin et al., 2015) and thus require further systematic investigation. However, only a few meta-analysis studies on the magnitude and variability of wheat yield and resource use efficiency (i.e., WP_*c*_ and NUE_*f*_) have been reported in China.

China has an incredibly diverse topography and highly variable soil and climatic conditions. Thus, wheat production in different regions varies significantly. Wheat is cultivated broadly in China, although the importance of wheat to the local economy differs by region (Huang et al., 2015). Thus, we hypothesized that the water saving potential (WSP) and N saving potential (NSP) varies with regions since environmental and agronomic factors play critical roles in the yield and resource use efficiency of wheat. In contrast, the complex interplay among these factors could substantially impact the trends in yield, WP_*c*_, and NUE_*f*_ (Lollato et al., 2019; Sidhu et al., 2019; Li et al., 2020). In addition, life cycle assessment (LCA) can examine the environmental impacts, including raw material extraction and transportation, agrochemical production and transportation, and arable farming in the field (Rebitzer et al., 2004), which can well assess subsequent environmental impact caused by various inputs of water and fertilizer in different regions of China. Thus, this study aimed to (1) investigate the effects of water and N inputs on yield, WP_*c*_, and NUE_*f*_ of wheat; (2) evaluate the extent of WSP and NSP based on the identified optimal input level and the environmental impact caused by various inputs of water and fertilizer in different regions using LCA; (3) evaluate, model, and rank the impacts of many environmental and agronomic factors on wheat yield, WP_*c*_, and NUE_*f*_ using structural models and decision tree-based importance ranking.

## Materials and Methods

### Literature Selection and Data Extraction

Literature and data selection criteria were similar to those used by Li et al. (2020; Supplementary Material). Ultimately, 126 studies, including 1,020 yield, 437 WP_*c*_, and 82 NUE_*f*_ paired observations were included in this study. In addition, information on study location, climatic conditions [mean annual temperature (MAT) and mean annual precipitation (MAP)], and soil properties [initial soil nutrient concentrations, potassium (K), and phosphorus (P)] were collected.

**FIGURE 1:**
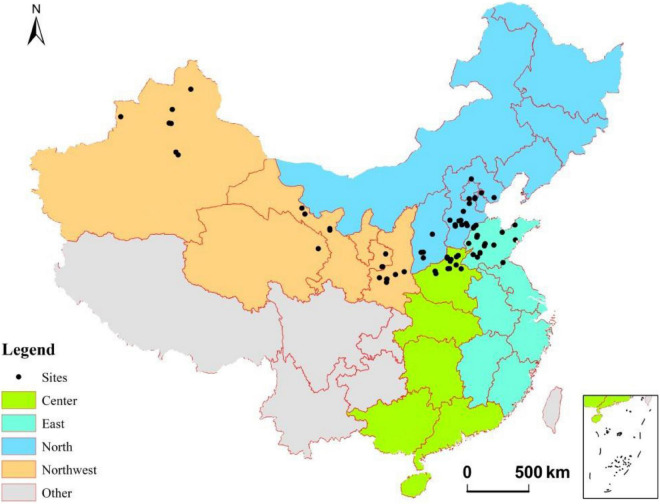
The geographical coverage of the studies used in the meta-analysis. Sites indicate studies’ location included in the current study. Refer to the details of regions classification in section “Literature Selection and Data Extraction”.

In addition, four regions, namely, Northwest (Gansu, Ningxia, Qinghai, Shaanxi, and Xinjiang provinces), North (Beijing, Jilin, Hebei, Heilongjiang, Inner Mongolia, Liaoning, Shanxi, and Tianjin provinces), Center (Guangdong, Guangxi, Henan, Hubei, and Hunan provinces), and East (Anhui, Fujian, Jiangsu, Jiangxi, Shandong, Zhejiang, and Shanghai provinces), were assigned as Zhang et al. (2017; Figure 1), while this classification excluded regions that have no studies.

### Data Process

#### Definitions

Water productivity (kg m^–3^) was calculated by the following equation:


(1) $$\text{WPc}=\frac{Y}{E\,T}$$

where *Y* is yield (kg ha^–1^) and ET is the total evapotranspiration (mm) (Ibragimov et al., 2007).

Fertilizer nitrogen use efficiency (kg kg^–1^) was calculated to compute *Y* over the total amount of nitrogen fertilizer applied in each hectare (N, kg ha^–1^) as follows:


(2) $$\text{NUEf}=\frac{Y}{N}$$

Water saving potential and NSP (Qin et al., 2016; Li et al., 2019) were calculated to the magnitude of the total water (the amount of water input, including precipitation and irrigation, in mm) and N savings, and WSP (mm, absolute value) was determined as follows:


(3) $$\mathrm{WSP}=W^{+}-W_{o\,p\,t}$$

where *W*^+^ represents the above-optimal water input, while W_*opt*_ indicates the optimal water input level producing the highest yield in a specific region.

The relative WSP (%) was calculated as follows:


(4) $$\mathrm{WSP}=\left(\frac{W^{+}-W_{o\,p\,t}}{W_{m\,a\,x}}\right)\,\text{×}\,100\%$$

where *W*_*max*_ represents the greatest *W*^+^. Similarly, NSP (kg ha^–1^, absolute value) was defined as follows:


(5) $$\mathrm{NSP}=N^{+}+-N_{o\,p\,t}$$

where *N*^+^ is the above-optimal N input, and N_*opt*_ is the optimal N input level producing the most significant yield in a specific region.

The relative NSP (%) was computed by the following equation:


(6) $$\mathrm{NSP}=\left(\frac{N^{+}-N_{o\,p\,t}}{N_{m\,a\,x}}\right)\,\text{×}\,100\%$$

where *N*_*max*_ stands for the greatest *N*^+^.

In addition, water saving value (WSV) and nitrogen saving value (NSV) were calculated based on the WSP and NSP in each region and, consequently, economic benefits were computed based on the price of water and fertilizer inputs in each region (Supplementary Table 1).

#### Effects of Independent Variables

The yield (*RR*_Y_), WP_c_ (*RR*_WPc_), and NUE_f_ (*RR*_*NUE*f_), the natural logarithm of the treatment over the control, were computed (Osenberg et al., 1999).


(7) $$R\,R_{\text{Y}}=\ln\left(\frac{Y_{\text{t}}}{Y_{\text{c}}}\right)=\ln\left(Y_{\text{t}}\right)-\text{ln}\,\left(Y_{\text{c}}\right)$$

Similarly, *RR*_*WPc*_ and *RR*_*NUEf*_ were computed as follows:


(8) $$R\,R_{\text{WPc}}=\ln\left(\frac{W\,P_{\text{t}}}{W\,P_{0}}\right)=\ln\left(W\,P_{\text{t}}\right)-\text{ln}\,\left(W\,P_{0}\right)$$

(9) $$R\,R_{\text{NUEf}}=\ln\left(\frac{N\,U\,E_{\text{t}}}{N\,U\,E_{0}}\right)=\ln\left(N\,U\,E_{\text{t}}\right)-\text{ln}\,\left(N\,U\,E_{0}\right)$$

where *Y*_*t*_, WP*_*t*_*, and NUE*_*t*_* represent the observed yield, WP_*c*_, and NUE_*f*_ with irrigation and/or N fertilization in a specific year of a study, respectively, while *Y*_*c*_, WP_0_, and NUE_0_ represent the yield, WP_*c*_, and NUE_*f*_ without irrigation and/or N fertilization, respectively. Subsequently, the weighted response ratio (effect size) and 95% confidence interval (CI) were calculated as described by Li et al. (2019). To facilitate the explanation, percentage change was calculated as [exp (*RR*_++_) − 1) × 100%.

Meta-analyses for each subgroup were processed based on the region and the water and N input level (refer to section “Definitions”). This was performed with the “metafor” package, using the restricted maximum likelihood estimator (RMLE) in the rma.uni model (Viechtbauer, 2010). Study ID was set as a random effect. The mean effect size and its 95% CI were calculated with bias correction generated by bootstrapping (4,999 iterations, Supplementary Tables 1-6).

The structural equation model (SEM) was also used to disentangle indirect and direct effects of climate (MAT), soil properties [initial soil organic carbon (SOC), available potassium (AK), and available phosphorus (AP)], and management practices (water and N inputs) on *RR*_*Y*_, *RR*_*WPc*_, and *RR*_*NUEf*_ using the “lavaan” package (Rosseel, 2012). *A priori* regression analyses were established based on the known effects and relationships among the variables (Grace, 2006).

#### Decision Tree-Based Modeling

A systematic decision tree-based modeling task was also implemented in MATLAB Programing Language (The MathWorks Inc., 2017, Natick, MA, United States) to evaluate the accuracy and feasibility of constructing data analytic models for predicting target variables: *RR*_*Y*_, *RR*_*WPc*_, and *RR*_*NUEf*_ using different ecophysiological variables observed across all included studies. Particularly, the values of 39 explanatory variables and features were manually extracted from each study (Supplementary Table 7). Following that, a 10-fold cross-validation (10-fold CV) training and testing paradigm was used to test modeling accuracy. The 10-fold CV routine randomly divided the entire dataset into 10 subsets of similar size. Each time, a decision tree-based prediction model was constructed based on nine subsets to test the remaining ones, and this process was repeated 10 times until all subsets of data were tested. All 39 features were also ranked according to the estimated predictor importance values calculated by summing changes in the risk due to the corresponding splits imposed on each predictor divided by the number of branch nodes.

#### Life Cycle Assessment

Life cycle assessment was used to evaluate the environmental impact caused by various inputs of water and fertilizer in different regions (Rebitzer et al., 2004). The evaluation scope of the LCA included the production, transportation, as well as emission and leaching due to fertilizer application in the field, while it did not involve the production and use of irrigation facilities, machinery, and equipment.

Eight environmental impact categories’ characterization was used in the study, namely, global warming potential (GWP), primary energy demand (PED), acidification potential (AP), abiotic depletion potential (ADP), eutrophication potential (EP), respiratory organics (RI), photochemical oxidative formation (POFP), and ecotoxicity (ETx). The characteristic factors of ADP, AP, and EP indicators were from the CML2002 model, and GWP and RI referred to the IPCC 2007 report, the IMPACT2002+ model, and the ReCiPe model (Goedkoop et al., 2009). The lists of water and fertilizer input in each region and the field emission coefficient of pollutants with fertilizer supply stemmed from the literature review. The background data of fertilizer production come from the Chinese life cycle database (CLCD).

#### Economic Analysis

Given the calculated WSP, NSP, and subsequent C emissions per hectare, and the prices of irrigation, N fertilizer, carbon price, and area of irrigated wheatland in different regions, the economic benefits of water and N saving per hectare and the total saving benefits of wheat production in a specific irrigated region were calculated. The irrigation price cannot accurately reflect its real value due to various subsidies in each region, and the value of N fertilizer was calculated according to the market price in different regions in 2019. The carbon price was the average price of the main carbon trading markets in different regions in 2019 (Center: Guangzhou, East: Shanghai, North: Beijing, North: Chongqing).

## Results

### Yields, Water Productivity, and Fertilizer Nitrogen Use Efficiency With Water and Nitrogen Addition

**FIGURE 2:**
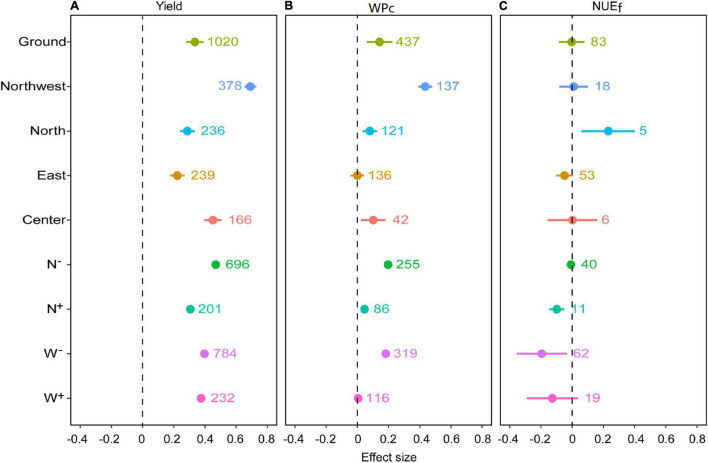
The effect size of the total water and nitrogen inputs on **(A)** grain yield, **(B)** water productivity (WP_*c*_), and **(C)** fertilizer nitrogen use efficiency (NUE_*f*_) of wheat. Effect size indicates the weighted response ratio of the treatment relative to the control and CI indicates the 95% CIs. The sample size of each variable was displayed adjacent to each bar. Particularly, the total effect size (ground) was classified by regions (check the detail of classification in Figure 1), and levels of water and nitrogen input. The water levels that were above or below optimal water input were defined as above-optimal (N^+^) and below-optimal N inputs (N^–^); and above-optimal (W^+^) and below-optimal water inputs (W^–^). Subcategories were indicated by colors.

Overall, *RR*_*Y*_ and *RR*_*WPc*_ were calculated as 40 and 15% with the input of water and N, respectively (*P* < 0.05, Figure 2). Regardless of regions, the input level of water or N, water, and N input enhanced *RR*_*Y*_. The *RR*_*Y*_ in the northwest of China was higher than that in the north, east, and center by 141, 211, and 53%, respectively (*P* < 0.05). The *RR*_*Y*_ in the center of China was significantly higher than that in the north and east of China by 57 and 103%, respectively. N^+^ reduced *RR*_*Y*_ by 53% compared with N^–^ (*P* < 0.05).

Water and N input significantly increased *RR*_*WPc*_ in the northwest, north, and center of China by 55, 8, and 11%, respectively (Figure 2). The *RR*_*WPc*_ in northwest of China was higher than that in the north and center of China by 444 and 327%, respectively (*P* < 0.05). Water and N input increased *RR*_*WPc*_ regardless of the N input levels. N^+^ reduced *RR*_*WPc*_ more than N^–^ by 328% (*P* < 0.05). W^–^ increased *RR*_*WPc*_ by 20% relative to control (*P* < 0.05).

Compared with control, water and N input significantly increased the effect size of *RR*_*NUEf*_ in the north of China by 26% (Figure 2), while N^+^ and W^–^ reduced *RR*_*NUEf*_ by 9 and 18%, respectively (*P* < 0.05).

### Water Saving Potential and Nitrogen Saving Potential With Water and Nitrogen Addition

The WSP had an overall mean of 11% (Table 1) and ranged from 0 to 57%, indicating that water input could be saved by up to 57% without significantly reducing wheat yield. However, a large variation was observed in the WSP values of different regions. For example, the WSP of center of China ranged between 0 and 31% (mean = 11%), and between 0 and 26% (mean = 10%) in the northwest of China. In the east and north of China, however, the WSP values ranged between 0–50 and 0–57%, respectively. Subsequently, water input enhanced yield when water input was lower than that of optimal level in the east (*R*^2^ = 0.08) and north of China (*R*^2^ = 0.08, *P* < 0.001, Table 2). However, an increase in water input significantly decreased yield when the water input was higher than that of optimal level in the east (*R*^2^ = 0.13) and north of China (*R*^2^ = 0.03, *P* < 0.01).

**TABLE 1:** Estimated mean water saving potentials (WSPs) of a specific region (for details, see Figure 1).

Region	*n*	OPTW (mm)	N input (kg ha^–1^)	Yield (t ha^–1^)	WP_*c*_ (kg m^–3^)	NUE_*f*_ (kg kg^–1^)	WSP (mm)			WSP (%)		
							Mean	Min	Max	Mean	Min	Max
Center	201	316	205	7.4	2.1	27.1	49	0	141	11	0	31
East	292	359	197	7.6	1.9	31.6	95	0	352	13	0	50
North	404	289	191	6.5	1.7	33.2	73	0	385	11	0	57
Northwest	424	557	322	5.1	1.5	26.3	76	0	192	10	0	26
Average	330	380	229	6.7	1.8	30.0	73	0	268	11	0	41

*Optimal total water input (OPTW) represents the minimal water input that produced maximum yields of a corresponding region. N input, grain yield, water productivity (WP_c_), and fertilizer nitrogen use efficiency (NUE_f_) represent the values associated with the optimal water input, respectively.*

**TABLE 2:** Linear regression model of the effects of the input level of water and nitrogen (N) on yield, water productivity (WP_*c*_), and N use efficiency (NUE_*f*_).

Dependent variable	Input level	Region	Equation	*R* ^2^	*P*
Yield (t ha^–1^)	W^–^	Total	*y* = 3.99*x* + 3.39	0.19	<0.001
		East	*y* = 2.14*x* + 5.63	0.08	<0.001
		North	*y* = 3.07*x* + 3.78	0.13	<0.001
		Northwest	*y* = 3.10*x* + 3.22	0.10	<0.001
	W^+^	Total	*y* = −1.07*x* + 8.28	0.03	0.004
		East	*y* = −3.07*x* + 11.5	0.18	<0.001
		North	*y* = −0.75*x* + 7.65	0.03	0.02
WP_*c*_ (kg m^–3^)	W^–^	Total	*y* = 0.16*x* + 1.30	0.05	<0.001
		East	*y* = −1.04*x* + 2.64	0.20	0.002
		Northwest	*y* = 0.94*x* + 0.99	0.10	<0.001
	W^+^	Total	*y* = −0.70*x* + 2.58	0.09	<0.001
		East	*y* = −0.39*x* + 2.13	0.14	0.04
		North	*y* = −0.91*x* + 2.81	0.19	<0.001
NUE_*f*_ (kg kg^–1^)	W^–^	Total	*y* = 15.81*x* + 18.20	0.05	0.005
		North	*y* = 30.78*x* + 6.67	0.12	0.008
		Northwest	*y* = 35.30*x*−0.20	0.21	<0.001
Yield (t ha^–1^)	N^–^	Total	*y* = 3.22*x* + 4.02	0.15	<0.001
		East	*y* = 1.62*x* + 6.01	0.05	0.002
		North	*y* = 1.81*x* + 4.98	0.07	<0.001
		Northwest	*y* = 3.00*x* + 3.27	0.12	<0.001
WP_*c*_ (kg m^–3^)	N^–^	Center	*y* = −1.78*x* + 2.97	0.30	<0.001
		Northwest	*y* = 0.98*x* + 1.02	0.15	<0.001
NUE_*f*_ (kg kg^–1^)	N^–^	Total	*y* = −13.17*x* + 41.72	0.04	0.01
		East	*y* = −19.59*x* + 46.58	0.23	0.004
		North	*y* = −26.28*x* + 55.89	0.14	0.007

**FIGURE 3:**
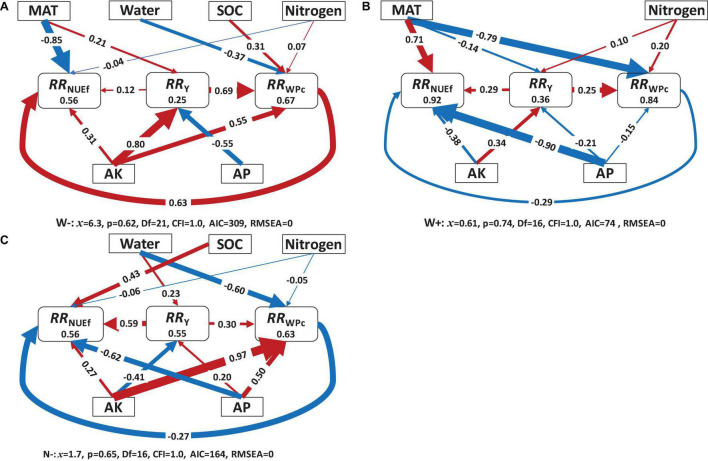
Structural equation model showing the direct and indirect effects of environmental and management conditions on the response ratio of yield (*RR*_*Y*_), water productivity (*RR*_*WPc*_), and fertilizer nitrogen use efficiency (*RR*_*NUEf*_) under **(A)** below-optimal water inputs (W^–^), **(B)** above-optimal water inputs (W^+^), and **(C)** below-optimal N inputs (N^–^). Results under the above-optimal N inputs (N^+^) were not shown due to data paucity. Numbers in the box indicate the variance explained by the model (*R*^2^). Numbers at arrows are standardized path coefficients. The width of the line indicates relative statistical significance (the thicker the more significant). MAT, mean annual temperature; Water, total water input; Nitrogen, total nitrogen inputs; SOC, initial soil organic carbon concentration; AK, initial soil available potassium; AP, initial soil available phosphorus.

*Levels of water and N input were classified based on optimal input level of water and N and data visualization (see Figure 3). See details of regions classification in section “Literature Selection and Data Extraction”.*

The NSP of all regions ranged from 0 to 62% (mean = 10%, Table 3), suggesting that N input could be reduced by up to 62% without largely reducing wheat yield. Large variations in the NSP of different regions were also observed. For instance, NSP ranged from 0 to 33% with means of 12 and 0–3% in the center and east of China, respectively. The NSP for the north and northwest of China ranged from 0 to 62% with means of 13 and 0–11%, respectively. Thus, an increase in N input enhanced yield when water input was lower than that of the optimal level in the north (*R*^2^ = 0.07) and northwest of China (*R*^2^ = 0.12, *P* < 0.01, Table 2).

**TABLE 3:** Estimated mean nitrogen saving potentials (NSPs) of a specific region (for details, see Figure 1).

Region	*n*	OPTN (kg ha^–1^)	Water input (mm)	Yield (t ha^–1^)	WP_*c*_ (kg m^–3^)	NUE_*f*_ (kg kg^–1^)	NSP (kg ha^–1^)			NSP (%)		
							Mean	Min	Max	Mean	Min	Max
Center	201	240	257	7.4	2.1	27.1	44	0	120	12	0	33
East	292	240	308	7.6	1.9	31.6	10	0	60	3	0	20
North	404	195	293	6.5	1.7	33.2	68	0	314	13	0	62
Northwest	424	270	322	5.1	1.5	26.3	60	0	255	11	0	49
Average	330	236	295	6.7	1.8	30	46	0	187	10	0	41

*Optimal total nitrogen input (OPTN) represents the minimal water input that produced maximum yields of a corresponding region. Water input, grain yield, water productivity (WP_c_), and fertilizer nitrogen use efficiency (NUE_f_) represent the values associated with the optimal N input, respectively.*

### Effect of Levels of Water and Nitrogen Input

The water input enhanced yield (*R*^2^ = 0.19) and WP_*c*_ (*R*^2^ = 0.05, *P* < 0.05, Table 2) when water input was lower than that of optimal level (Supplementary Figures 1A,B). Overuse of water decreased wheat yield (*R*^2^ = 0.03) and WP_*c*_ (*R*^2^ = 0.09, *P* < 0.05). In addition, the increase in water input enhanced NUE_*f*_ (*R*^2^ = 0.05) when water input was lower than that of optimal level (Supplementary Figure 1C, *P* < 0.05).

The N input increased yield (*R*^2^ = 0.15) and NUE_*f*_ (*R*^2^ = 0.04, *P* < 0.05, Table 2) when N input was lower than that of optimal level (Supplementary Figures 1D,F). However, there were no clear patterns for the effect of the input level of N on yield, WP_*c*_, or NUE (Supplementary Figures 1D-F).

### Factors Affecting Response Ratio of Yield, Response Ratio of WP_*c*_, and Response Ratio of NUE_*f*_ Identified by Structural Equation Model

For relationships among *RR*_*Y*_, *RR*_*WPc*_, and *RR*_*NUEf*_ and various environmental and agronomic factors (Figure 3), no correct converging model was identified for N^+^. For W^–^, there was a positive correlation among *RR*_*Y*_, *RR*_*WPc*_, and *RR*_*NUEf*_. Meanwhile, AK (positive) and AP (negative) affected *RR*_*Y*_ substantially (*P* < 0.01), and 25% of the variation in *RR*_*Y*_ was explained (*P* < 0.05, Figure 3A). *RR*_*WPc*_ was positively impacted by AK (0.55), followed by SOC (0.31), and water input (−0.37), with 67% of the variation in *RR*_*WPc*_ explained (*P* < 0.001). However, *RR*_*NUEf*_ was negatively affected by MAT (−0.85), followed by *RR*_*WPc*_ (0.63), and AK (0.31), with 56% of the variation in *RR*_*NUEf*_ explained (*P* < 0.05).

For W^+^, 36% of *RR*_*Y*_ variation was explained by AK, while N input, AP, and MAT, none of them, were significant (*P* > 0.4, Figure 3B). *RR*_*WPc*_ was positively impacted by *RR*_*Y*_ (0.25) and N input (0.20), but negatively affected by MAT (−0.79) and water input (−0.37), with 84% of the variation in *RR*_*WPc*_ explained (*P* < 0.05). However, *RR*_*NUEf*_ was negatively affected by AP (−0.90), followed by AK (−0.38) and *RR*_*WPc*_ (−0.29), and positively affected by MAT (0.71) and *RR*_*Y*_ (0.29), with 92% of the variation in *RR*_*NUEf*_ explained (*P* < 0.001).

For N^–^, *RR*_*Y*_ was positively affected by water input (0.23), with 55% of the variation in *RR*_*Y*_ explained (*P* < 0.001, Figure 3C). *RR*_*WPc*_ was positively impacted by *RR*_*Y*_ (0.30), AK (0.97), and AP (0.50), but negatively affected by water input (−0.60), with 63% of the variation explained (*P* < 0.001). Meanwhile, *RR*_*NUEf*_ was negatively affected by AP (−0.62), followed by *RR*_*WPc*_ (−0.27), but positively affected by *RR*_*Y*_ (0.59), and SOC (0.43), with 92% of the variation explained (*P* < 0.001).

### Life Cycle Assessment of Water and Nitrogen Input

Given the scaled wheatland, for a hectare of wheatland, LCA indicators resulting from water and N input in northern China were higher than that in the rest of the regions (Table 4), followed by the central and eastern China, and the lowest was in the northwest. Particularly, the GWP in central, eastern, and northwestern China was 92, 91, and 87% of that in northern China.

**TABLE 4:** Effect of nitrogen and water input on environmental indexes in a specific region (for details, see Figure 1).

Area	GWP	GWP per T	PED	PED per T	AP	AP per T	ADP	ADP per T	EP	EP per T	RI	RI per T	POFP	POFP per T	ETx	Etx per T
Center	1634.37	220.86	17087.05	2309.06	9.69	1.31	0.04	0.0054	3.66	0.49	1.73	0.23	2.04	0.28	383.99	51.89
East	1620.24	213.19	17030.01	2240.79	9.53	1.25	0.04	0.0053	3.50	0.46	1.75	0.23	1.94	0.26	360.04	47.37
North	1772.94	272.76	19386.30	2982.51	10.53	1.62	0.04	0.0062	3.66	0.56	1.99	0.31	2.21	0.34	407.90	62.75
Northwest	1541.81	302.32	16839.85	3301.93	9.14	1.79	0.04	0.0078	3.16	0.62	1.73	0.34	1.90	0.37	349.76	68.58

*Indexes include global warming potential (GWP; kg CO_2_ eq), primary energy demand (PED; MJ), acidification potential (AP; kg SO_2_ eq), abiotic depletion potential (ADP; kg antimony eq), eutrophication potential (EP; kg P_3_O_4_ eq), respiratory inorganics (RI; kg PM2.5 eq), photochemical oxidant formation (POFP; kg NMVOC eq), and ecotoxicity (ETx; CTUe).*

Based on the scaled wheat yield, for a one-tone yield (Table 4), LCA indicators resulting from water and N input in northwest China were higher than that in other regions. The GWP of the north, central, and eastern China was 90, 73, and 70% of that in northern China.

### Economic Analysis of Water and Nitrogen Input

The irrigation cost varied greatly in regions of China (Table 5). The irrigation cost was higher in northwest and northern China, and the lowest one was in eastern China. The carbon price in the north was the highest, about 10 times higher than that in the northwest. The reduction of greenhouse gas emissions due to saved water consumption was highest in eastern China and lowest in central China (Table 5). For the mitigation of greenhouse gas emissions by reducing N fertilizer application, the north was more prominent and the east was the lowest (Table 6). Thus, the saved benefit per hectare of wheatland was mainly caused by enhanced values of water saving and reduction of carbon emission. Specifically, the northwest with higher irrigation prices had the highest water saving benefit, followed by northern and eastern China. The northwest and north could have up to 0.51 and 0.45 billion yuan WSVs, respectively. Northern China had the highest carbon reduction value because of its high carbon price.

**TABLE 5:** Estimated mean water saving (WS) or water saving value (WSV) and corresponding economic benefits in a specific region (for details, see Figure 1).

	Irrigation (Yuan)	WS (mm)	C price (Yuan t^–1^)	GWP save (kg CO_2_ ha^–1^)	WSV (Yuan)	GWP SV (Yuan)	Total WS (Yuan ha^–1^)	GWPSV/WS (%)	Irrigation area (ha)	Region WSV (Yuan)
Center	0.30	49	20.51	93.34	147.00	1.91	148.91	1.30	2,102,596	313,097,570
East	0.15	95	29.80	180.97	142.50	5.39	147.89	3.78	2,227,713	329,456,476
North	0.42	73	61.98	139.06	306.60	8.62	315.22	2.81	1,422,983	448,552,701
Northwest	0.46	76	6.11	144.78	349.60	0.88	350.48	0.25	1,464,135	513,150,035

*See details of water saving in Table 1.*

**TABLE 6:** Estimated mean nitrogen saving (NS) or nitrogen saving value (NSV) and corresponding economic benefits in a specific region (refer to Figure 1).

	N (Yuan ha^–1^)	NS (kg)	C price (Yuan t^–1^)	GWP save (kg CO_2_ ha^–1^)	NSV (Yuan)	GWP SV (Yuan)	Total NS (Yuan)	GWPSV/NS (%)	Wheat area (ha)	Region NSV (Yuan)
Center	4.48	44	20.51	131.86	197.12	2.70	199.82	1.37	5,673,670	1,133,712,739
East	4.13	10	29.80	29.97	41.30	0.89	42.19	2.15	3,934,430	165,993,601
North	4.30	68	61.98	179.81	292.40	11.14	303.54	3.81	2,764,270	839,066,516
Northwest	4.31	60	6.11	137.85	258.60	0.84	259.44	0.32	2,929,640	760,065,802

*Refer to the details of nitrogen saving in Table 3.*

### Factors Affecting Response Ratio of Yield, Response Ratio of WP_*c*_, and Response Ratio of NUE_*f*_ Identified by Decision Tree-Based Regression

**FIGURE 4:**
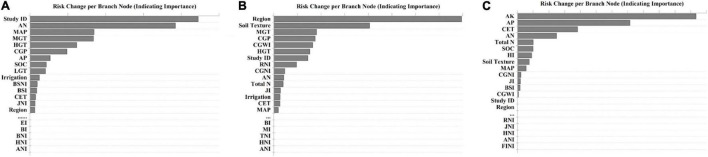
Feature ranking (top-15 and bottom-5) based on risk change per branch node in modeling response ratios of **(A)** yield (*RR*_*Y*_), **(B)** water productivity (*RR*_*WPc*_), and **(C)** fertilizer nitrogen use efficiency (*RR*_*NUEf*_) using decision tree-based algorithms. Refer to Table 1 for detailed information on each variable’s abbreviation.

Based on risk change per branch node of each explanatory variable and feature, the regression models ranked all features, indicating their importance in affecting the response variable (*RR*), and the top-15 and bottom-5 features were identified (Figure 4). As indicated, the site-specific information, such as study identification, climatic conditions, initial soil nutrient, and physical condition, tend to have a greater impact on modeling accuracy. Interestingly, split N application at different growth stages appeared to be the least informative feature affecting yield, WP_*c*_, and NUE_*f*_.

## Discussion

### Water and Nitrogen Input Significantly Increased Wheat Yield and Water Productivity

The present national-scale meta-analysis revealed that water and N input significantly increased wheat yield and WP_*c*_, by averages of 40 and 15%, respectively. We found that the overall mean WSP and NSP of China are 11 and 10%, respectively. This study indicated that the water and N input levels affect wheat yield and resource use efficiency, both of which varied considerably among regions. This finding partially agreed with what was reported by Liu et al. (2020) based on a meta-analysis focusing on the North China Plain but also emphasized that similar trends can also be found in other wheat production regions in China. Soil nutrient status (e.g., AP, AK, and AN) and SOC concentration were found more crucial than water and N management practices or climate (MAT) in determining yield, WP_*c*_, and NUE_*f*_; besides, their effects broadly interact with the water and N input levels. Overall, the effect size of water and N on the yield of northwest China was greater than in any other regions. This observation might be explained by the fact that the majority of the northwest China region is dominated by a semiarid and arid environment with salty soils and great dependency on irrigation water input (Zhang et al., 2014). Thus, the combined effects of salinity reduction and enhanced N use efficiency contributed by irrigation water could result in greater effect sizes compared with other temperate or humid areas included in this study.

Regardless of region and input level, the input of water and N during wheat production increased the yield potential. In addition, northwest and central China had greater yields than north and eastern China. This yield disparity could be attributed to differences in climatic conditions or irrigation methods (Supplementary Figure 2). For example, center of China features a humid climate, small fields, and furrow irrigation, which are not common in arid and semiarid environments such as northwest China. Similarly, increases in WP_*c*_ due to water and N input were highest in northwest China. This result is likely due to the practice of soil mulching with plastics (Ma et al., 2018) or straws (Qin et al., 2015), which is prevalent in the region. For example, using 1,278 observations in northwestern China, Ma et al. (2018) found that mulching with plastic files increased wheat yield and WP_*c*_ by about 20 and 22%, respectively. Due to data scarcity, however, this study did not explore soil mulching to depth and could, thus, not make a further conclusion on this study. N^–^ resulted in more yield increase and higher WP_*c*_ than N^+^, which is in line with NSP results. This finding has important implications for optimizing N fertilization during wheat production in China, considering the current high utilization of N fertilizer. Similarly, water and N input increased WP_*c*_, except in the east of China, where high levels of water input are typical due to the relatively greater abundance of irrigation water and precipitation. While the input of both water and N increased NUE_*f*_ in the north of China, a high level of water input only could consistently decrease NUE_*f*_.

### Water Saving Potential and Nitrogen Saving Potential With Water and Nitrogen Addition

Water and N inputs did not continuously increase yield, WP_*c*_, or NUE_*f*_ (Supplementary Figure 3). According to Rathore et al. (2017), the positive effect of N input on NUE_*f*_ is primarily due to increased N availability and better crop growth (Rathore et al., 2017). In contrast, water or N overuse might induce excessive vegetative growth, limiting the partitioning of photosynthates toward the growth of reproductive tissues (Bennett et al., 1989). In addition, higher levels of N input could decrease NUE_*f*_ because of more significant losses (e.g., leaching and volatilization, among others) (Rathore et al., 2017). Soil water content can significantly affect wheat NUE_*f*_ by affecting soil N availability and plant N uptake (Ashraf et al., 2016). The addition of N also enhances wheat growth and thus increases WP_*c*_. Moreover, previous studies have shown that N addition in N-limited soils increases WP_*c*_ (Amir et al., 1991; Qiu et al., 2008; Cossani and Sadras, 2018). Moreover, the LCA indicated that the environmental impact caused by water and N inputs varied with regions (Table 4), particularly N input, which implies that appropriate water and N input should be further encouraged to alleviate environmental burns in the process of wheat production. The north region yielded higher environmental indexes that might attribute to N application rate, irrigation times, and seasonal precipitation (Qiu et al., 2008; Liu et al., 2020). Our LCA results also indicated that irrigation and fertilization have a significant impact on environmental damages, such as GWP, emphasizing the need to decrease the intensity of input energy for sustainable wheat production (Taki et al., 2018).

The east and north of China had a relatively higher WSP and NSP, respectively. Given the overall high amount of water and N inputs in China, these WSP and NSP values indicate that sustainable water and N management practices could be achieved without impeding wheat grain yield in these areas. Optimizing water and N inputs should consider crop development, water, and nutrient demand holistically (Qin et al., 2016; Li et al., 2019), rather than water and N input alone, or even combined (Supplementary Figure 3). Regressions between the water and N input and yield, WP_*c*_, and NUE_*f*_ revealed a series of significant linear relations (*P* < 0.05). However, some of the *R*^2^ values were poor (between 0.04 and 0.23, Table 2). This finding indicates that both N and water have significant impacts on those response variables. Thus, improving sustainability on a cropping system level needs the comprehensive implementation of practices that consider a lot more than just N and water inputs alone (e.g., environmental and managerial interactions, and site-specific conditions and resources). According to our study, to further mitigate greenhouse gas emissions and reduce energy consumption from wheat production, focuses should be placed on promoting water-saving management in eastern and northwest China (less water and adequate N), and N-saving management in central and northern China (less N and adequate water input). While for cost-saving purposes, the benefits of water saving in eastern China should be considered, and N saving in central and northern China should be highlighted. The temperate and subtropical monsoon climate zones of wheat production areas in China (all areas except for Xinjiang, Inner Mongolia, and Gansu) are deemed more suitable for planting wheat in other areas in terms of energy efficiency and resource utilization because of the ample precipitation, sunlight, and superior soil conditions (Zhao et al., 2019). Combining the results from this study, more efficient irrigation systems and N fertilizers and application methods should be focused in the future to further boost the energy output from the center, east, and the bottom parts of the north and northwest regions in China. The high GWP and PED particularly in the north portion of the northern wheat production region might require more suitable crops from an energy and resource use efficiency perspective.

### Factors Affecting Response Ratio of Yield, Response Ratio of WP_*c*_, and Response Ratio of NUE_*f*_

As discussed earlier, this study demonstrated further investigation that soil nutrients (e.g., SOC, AP, AK, and AN) were more important than climate (MAT) or water and N management practices in determining wheat yield, WP_*c*_, and NUE_*f*_ (Figure 4 and Supplementary Table 8). The highlighted significance of improving soil property-related indices to improve productivity was consistent with prior findings. For instance, initial soil nutrients, such as AP and AK concentrations, were vital for increasing the actual yield, WP_*c*_, and NUE_*f*_, which could partly shadow the contributions of water and/N management practices (Lollato et al., 2019). This is because the production of grain crops, such as wheat, typically requires higher amounts of K and comparable levels of N, relative to biomass crops, as indicated in previous research (Lu et al., 2017). A recent study, synthesizing 155 long-term experiments, demonstrated that the application of P and K enhances wheat yield and NUE_*f*_ (Lollato et al., 2019). P deficiency reduces the number of spikes by limiting tiller formation, root biomass, and exploration of the soil profile (Fageria and Baligar, 1999), and thus reduce the wheat yields. A study in the Yangtze plains reported that the depletion of soil K could significantly reduce crop yield and nutrient use efficiency (Lu et al., 2017). However, as discussed earlier, overuse of water or N affects plant physiology and nutrient demand considerably, thus causing significant variation in SEM at various water or N input levels. Furthermore, although all presented overall SEM models are significant (indicated by non-significant Chi-square test statistics), results obtained from an individual path should not be over-interpreted because they might be insignificant (e.g., effects of AK, N input, AP, and MAT on *RR*_*Y*_). Focuses should only be placed on those with large *R*^2^ values and great significance levels while evaluated individually. Below- and above-optimal water input levels demonstrated very different responses between response and explanatory variables (Figure 3). Particularly, when water input was below the optimal range, the total water input level was critical in affecting the overall *RR*_*WPc*_, which indirectly affects *RR*_*Y*_ and *RR*_*NUEf*_. The negative coefficient could be caused by the fact there might exist additional limiting factors that hinder a positive response toward additional water input even at the suboptimal levels. When total water input was above the optimal level, the entire water input variable became insignificant and was, therefore, eliminated from the SEM model. Initial soil nutrient status (AK and AP) appeared to have a greater impact on *RR*_*Y*_ and *RR*_*WPc*_ when water input was below the optimal level than above. Finally, at the suboptimal N input level, total water input and SOC indicated a significant impact on *RR*_*WPc*_ and *RR*_*NUEf*_, respectively, greatly outweighing the effects contributed by N input itself. This indicates that long-term soil C building and alternation of soil hydrological property could potentially improve wheat yield and resource use efficiency more effectively than adding exogenous N inputs. Similarly, AK and AP indicated a great significance as cereal grain crops depend greatly on K and P for seed production in addition to N. This overall observation under the suboptimal N condition hints that rather than focusing on N inputs, greater consideration should be given to the overall soil physicochemical condition, particularly for improving productivity and sustainability of wheat systems.

Climatic conditions were shown to have a great impact on wheat yield and resource use efficiency (Supplementary Figure 2). For example, negative correlations between MAT and *RR*_*Y*_ or *RR*_*NUEf*_ were identified in this study (Figure 3), which agreed qualitatively with a previous study based on boundary-function analysis, indicating that the maximum yields for wheat are typically obtained at moderate MAT (Andrade and Satorre, 2015). Although high-temperature stress affecting crop physiology and productivity was well documented in previous research (Peng et al., 2004), few studies dissect the impacts of temperature responses according to growing season average, low, or peak values vs. the annual values. Particularly for wheat, its main growing season typically spans the cooler time of the year, thus, including summer temperatures (where extreme annual temperatures usually occur) might be unreasonable. Therefore, within-growing season variables (e.g., temperature and precipitation) should serve as better predictors (Mubeen et al., 2016) for conducting advanced data analytic modeling as they directly affect crop heat unit accumulation and development.

The adoption of decision tree-based regression in agricultural research is not new (Smidt et al., 2016). The reason for conducting decision tree-based modeling on *RR* is that the dataset collected for this particular meta-analysis had a great level of completeness (covering a large temporal and spatial scale) and resolution (e.g., inclusion of whole-year precipitation and temperature, initial soil nutrient condition, as well as key agronomic inputs, i.e., water and N, broke down by growth stages), thus, offering an excellent opportunity for applying more sophisticated data analytic algorithms rather than standard meta-analysis methods and ANOVA. The feature ranking results (Figure 4) provided findings that no individual study could thoroughly investigate alone. For example, study identification remained important, indicating that site-specific environmental and managerial factors (in addition to those already included in the modeling process) were very informative in determining *RR*_*Y*_ and *RR*_*WPc*_ (first and seventh ranking, respectively). Initial soil nutrient conditions, such as AN, AP, and AK, had a great impact on yield and NUE_*f*_ but not much on WP_*c*_. This finding agreed qualitatively with some other data synthesis type studies, indicating yield increases with more available soil nutrient contents (Hossard et al., 2016; Lollato et al., 2019); however, information relating to the effects of initial soil nutrients on WP_*c*_ or NUE_*f*_ alone is almost non-existent. For example, in another meta-analysis study, Liu et al. (2020) evaluated the effects of initial soil total N, pH, and bulk density on NUE_*f*_ and WP_*c*_. However, other key nutrients (e.g., P and K) were completely ignored, and this limitation was also acknowledged by the authors. This is largely caused by the fact that the initial soil nutrient condition is an intrinsic factor correlated with each experimental site, which cannot be randomly assigned and evaluated as a treatment factor (thus, treated as blocking factors). It is worth noticing that parameters, such as AN, AP, and AK, are completely different from pre-sowing nutrient inputs. This finding suggested that maintaining long-term soil health, which leads to enhanced intrinsic soil nutrient condition, is more important than instant nutrient inputs at the pre-planting stage or in the growing season. Similarly, Mustafa et al. (2021) reported that soil conservation practices are generally more effective in improving wheat yield than other strategies (e.g., variety selection, planting date, etc.); however, no systematic analysis was used to identify the key soil conservation factors for wheat production. The initial SOC indicated a greater impact on NUE_*f*_ and yield than WP_*c*_. Both average and peak growing-season temperatures (MGT and HGT) seemed to be very important in affecting both yield and WP_*c*_, shadowing the effects of MAT, which indicated the importance of adopting growing-season specific climatic information rather than focusing on just annual values (Addy et al., 2020).

We are aware of the importance of the precipitation and temperature during important phases of wheat growth; however, our study is badly limited owing to a paucity of such data. Thus, broader interpretations should be made with caution. In addition, we acknowledge that different wheat types (spring vs. winter) might have different response patterns toward variation in temperatures and frequency of frost events. While based on results from this study, wheat type indicated less impact on modeling accuracy. It is also worth noting that although the current dataset contains 89% of studies (112) using winter wheat and only 11% using spring wheat (14), significant impacts caused by wheat type should still be identified using robust mathematical models such as decision trees. Finally, we acknowledge that successful production of wheat requires other inputs such as herbicides, pesticides, P, and K. However, for this meta-analysis, we chose to focus on two of the most limiting inputs dominating wheat production in China (and globally), i.e., water and N. According to Deng et al. (2021), about 76 and 82% of greenhouse gas emission reduction and NUE enhancement could be achieved in the production phase of wheat out of the entire supply chain, respectively. Water and N are the most crucial inputs during the production phase of wheat farming.

## Conclusion

Meta-analysis indicated that irrigation and N addition increased the average yield and WP_*c*_ by 40 and 15%, respectively, relative to control treatments with no irrigation or fertilizer application. The mean WSP and NSP in China were estimated at 11 and 10%, respectively. In conjunction with modeling and LCA, this study indicated that soil nutrients and initial concentrations of P, K, and SOC affect yield, WP_*c*_, and NUE_*f*_ more significantly than climate (MAT) or water and N management practices. These effects are more pronounced at the below-optimal water and N levels than above. Maintaining long-term soil health, which leads to enhanced intrinsic soil nutrient condition at planting, is more important than N inputs during the production phase; to further mitigate greenhouse gas emissions, water- or N-saving management should be promoted, which will vary in different regions of China. These results could guide and inform the design and implementation of large-scale modeling studies on sustainable water and N management strategies for sustainable cropping system design and reduction in energy consumption for wheat production.

## Data Availability Statement

The original contributions presented in the study are included in the article/Supplementary Material, further inquiries can be directed to the corresponding authors.

## Author Contributions

ZL: conceptualization, investigation, formal analysis, visualization, and writing – original draft. SC, QZ, and YL: conceptualization, supervision, funding acquisition, and writing – review and editing. GX: software, writing – review and editing, and validation. QF and CC: writing – review and editing. All authors contributed to the article and approved the submitted version.

## Conflict of Interest

The authors declare that the research was conducted in the absence of any commercial or financial relationships that could be construed as a potential conflict of interest.

## Publisher’s Note

All claims expressed in this article are solely those of the authors and do not necessarily represent those of their affiliated organizations, or those of the publisher, the editors and the reviewers. Any product that may be evaluated in this article, or claim that may be made by its manufacturer, is not guaranteed or endorsed by the publisher.

## Abbreviations

ADP: abiotic depletion potential
AP: available phosphorus
AK: available potassium
CI: confidence interval
CLCD: Chinese life cycle database
EP: eutrophication potential
ET: total evapotranspiration
ETx: ecotoxicity
GWP: global warming potential
SOC: initial soil organic carbon
LCA: life cycle assessment
MAT: mean annual temperature
MAP: mean annual precipitation
N: nitrogen
NSPs: N saving potentials
NSV: nitrogen saving value
Nopt: optimal N input level
NUE_f_: N use efficiency
N^+^: above-optimal N inputs
N^–^: below-optimal N inputs
PED: primary energy demand
POFP: photochemical oxidative formation
RI: respiratory organics
*RR*: natural logarithm of the treatment over the control
*RR*_*NUEf*_: response ratio of NUE_f_
*RR*_WPc_: response ratio of WP_c_
*RR*_Y_: response ratio of yield
SEM: structural equation model
WP_c_: water productivity
WSPs: water saving potentials
WSV: water saving value
Wopt: optimal water input level.
