# Supplementary material for: Optimizing Wheat Yield, Water, and Nitrogen Use Efficiency With Water and Nitrogen Inputs in China: A Synthesis and Life Cycle Assessment
Source: Front Plant Sci. 2022 Jun 16;13:930484. doi: 10.3389/fpls.2022.930484 (PMC9244784; doi:10.3389/fpls.2022.930484)
Supplement: Supplementary file 1 [file Data_Sheet_1.DOCX]

**Table S1.** The price of water and fertilizer inputs in each region.

|  | Irrigation (Yuan m^-3^) | Urea (Yuan kg^-1^) | Nitrogen (Yuan kg^-1^) | Irrigation wheat area (ha) |
| --- | --- | --- | --- | --- |
| **Center** |  |  |  |  |
| Henan | 0.30 | 2.06 | 4.48 | 2,102,596 |
|  |  |  |  |  |
| **East** |  |  |  |  |
| Shandong | 0.15 | 1.90 | 4.13 | 2,227,713 |
|  |  |  |  |  |
| **North** |  |  |  |  |
| Liaoning | 0.18 | 2.16 | 4.70 | 1,183 |
| Hebei | 0.34 | 1.90 | 4.13 | 1,215,716 |
| Shanxi | 0.42 | 1.92 | 4.17 | 200,610 |
| Beijing | 0.75 | 1.94 | 4.22 | 5,474 |
|  |  |  |  |  |
| **Northwest** | |  |  |  |
| Xinjiang | 0.98 | 1.65 | 3.59 | 832,984 |
| Qinghai | 0.21 | 2.30 | 5.00 | 36,363 |
| Gansu | 0.25 | 2.03 | 4.41 | 241,287 |
| Ningxia | 0.35 | 1.97 | 4.28 | 43,722 |
| Shannxi | 0.50 | 1.96 | 4.26 | 309,779 |

**Table S2.** Heterogeneity (Q) and probability (*P*) among n observations of the effect of water and nitrogen inputs on grain yield, water use efficiency (WUE), nitrogen use efficiency (NUE) of wheat.

|  | Study size | Publication bias | Test of heterogeneity | | | Random effects model | |
| --- | --- | --- | --- | --- | --- | --- | --- |
|  |  | *Fail-safe number* | Q | n-1 | *p*-value | *z* | *p*-value |
| Yield | 95 | 14130939 | 41865 | 1019 | < .0001 | 11.7047 | <.001 |
| WUE | 49 | 304396 | 7082 | 436 | < .0001 | 3.3913 | <.001 |
| NUE | 12 | 353 | 680 | 82 | < .0001 | -0.0889 | <.001 |

**Table S3.** Heterogeneity (between group (*Q*_B_) and within group (*Q*_W_)) and probability (*P*) among n observations of effect of water and nitrogen inputs on grain yield, water use efficiency (WUE), nitrogen use efficiency (NUE) of wheat. Details of regional classification see section 2.2.

|  |  | Study size | Test of heterogeneity | | | | | Random effects model | |
| --- | --- | --- | --- | --- | --- | --- | --- | --- | --- |
|  |  |  | n | *Q*_B_ | *p*-value | *Q*_W_ | *p*-value | *z* | *p*-value |
| Yield | Center | 15 | 166 | 13718 | < .0001 | 28146 | < .0001 | 15.971 | <.001 |
|  | East | 33 | 239 |  |  |  |  | 9.439 | <.001 |
|  | North | 21 | 236 |  |  |  |  | 12.017 | <.001 |
|  | Northwest | 26 | 378 |  |  |  |  | 36.795 | <.001 |
| WUE | Center | 6 | 42 | 2005 | < .0001 | 5077 | < .0001 | 2.541 | 0.011 |
|  | East | 20 | 136 |  |  |  |  | -0.015 | 0.988 |
|  | North | 11 | 121 |  |  |  |  | 3.419 | <.001 |
|  | Northwest | 12 | 137 |  |  |  |  | 19.529 | <.001 |
| NUE | Center | 1 | 6 | 108 | < .0001 | 468 | < .0001 | 0.024 | 0.981 |
|  | East | 8 | 53 |  |  |  |  | -1.808 | 0.071 |
|  | North | 3 | 5 |  |  |  |  | 2.640 | 0.008 |
|  | Northwest | 2 | 18 |  |  |  |  | 0.214 | 0.831 |

**Table S4.** Heterogeneity (between group (*Q*_B_) and within group (*Q*_W_)) and probability (*P*) among n observations of effect of levels of water and nitrogen inputs on grain yield, water use efficiency (WUE), nitrogen use efficiency (NUE) of wheat. The water levels that were above or below optimal water input were defined as above-optimal and below-optimal water inputs, denoted as W^+^ and W^-^, respectively; and above-optimal and below-optimal N inputs were denoted as N^+^ and N^-^, respectively. Details of optimal water and nitrogen input see Table 1 and 2.

|  |  | Study size | Test of heterogeneity | | | | | Random effects model | |
| --- | --- | --- | --- | --- | --- | --- | --- | --- | --- |
|  |  |  | n | *Q*_B_ | *p*-value | *Q*_W_ | *p*-value | *z* | *p*-value |
| Yield | W+ | 45 | 232 | 39182 | < .0001 | 41848 | < .0001 | 76.806 | <.001 |
|  | W- | 86 | 784 |  |  |  |  | 182.2 | <.001 |
|  | N+ | 39 | 201 | 41887 | < .0001 | 39143 | < .0001 | 63.258 | <.001 |
|  | N- | 79 | 696 |  |  |  |  | 188.494 | <.001 |
| WUE | W+ | 25 | 116 | 2059 | < .0001 | 6155 | < .0001 | 0.743 | 0.458 |
|  | W- | 46 | 319 |  |  |  |  | 45.340 | <.001 |
|  | N+ | 15 | 86 | 414 | < .0001 | 6668 | < .0001 | 8.044 | <.001 |
|  | N- | 35 | 255 |  |  |  |  | 40.923 | <.001 |
| NUE | W+ | 7 | 19 | 15 | < .0001 | 560 | < .0001 | -1.5139 | 0.1300 |
|  | W- | 12 | 62 |  |  |  |  | -1.164 | 0.0179 |
|  | N+ | 3 | 11 | 20 | < .0001 | 555 | < .0001 | -4.013 | <.001 |
|  | N- | 8 | 40 |  |  |  |  | -0.598 | 0.550 |

**Table S5.** Heterogeneity (between group (*Q*_B_) and within group (*Q*_W_)) and probability (*P*) among n observations of effect of aridity index (AI) on grain yield, water use efficiency (WUE), nitrogen use efficiency (NUE) of wheat.

|  | AI | Study size | Test of heterogeneity | | | | | Random effects model | |
| --- | --- | --- | --- | --- | --- | --- | --- | --- | --- |
|  |  |  | n | *Q*_B_ | *p*-value | *Q*_W_ | *p*-value | *z* | *p*-value |
| Yield | Arid | 4 | 54 | 1286 | < .0001 | 36157 | < .0001 | 6.280 | <.001 |
|  | Semi-arid | 10 | 81 |  |  |  |  | 9.531 | <.001 |
|  | Semi-humid | 48 | 534 |  |  |  |  | 24.013 | <.001 |
|  | Humid | 29 | 316 |  |  |  |  | 23.992 | <.001 |
| WUE | Arid | 3 | 36 | 175 | < .0001 | 6496 | < .0001 | 5.895 | <.001 |
|  | Semi-arid | 1 | 10 |  |  |  |  | 7.283 | <.001 |
|  | Semi-humid | 23 | 227 |  |  |  |  | 7.118 | <.001 |
|  | Humid | 19 | 139 |  |  |  |  | 5.906 | <.001 |
| NUE | Arid | - | - | 19 | < .001 | 353 | < .0001 |  | <.001 |
|  | Semi-arid | 1 | 10 |  |  |  |  | -2.159 | 0.031 |
|  | Semi-humid | 4 | 27 |  |  |  |  | -0.245 | 0.605 |
|  | Humid | 7 | 45 |  |  |  |  | -3.691 | 0.081 |

**Table S6.** Heterogeneity (between group (*Q*_B_) and within group (*Q*_W_)) and probability (*P*) among n observations of effect of irrigation methods on grain yield, water use efficiency (WUE), nitrogen use efficiency (NUE) of wheat.

|  | Method | Study size | Test of heterogeneity | | | | | Random effects model | |
| --- | --- | --- | --- | --- | --- | --- | --- | --- | --- |
|  |  |  | n | *Q*_B_ | *p*-value | *Q*_W_ | *p*-value | *z* | *p*-value |
| Yield | Drip | 9 | 60 | 1226 | < .001 | 41676 | < .001 | 7.465 | 0.390 |
|  | Furrow | 39 | 375 |  |  |  |  | 17.923 | <.001 |
|  | Sprinkling | 8 | 57 |  |  |  |  | 8.897 | 0.094 |
| WUE | Drip | 2 | 14 | 149 | < .001 | 6953 | < .001 | 0.859 | 0.390 |
|  | Furrow | 21 | 184 |  |  |  |  | 5.496 | <.001 |
|  | Sprinkling | 7 | 51 |  |  |  |  | 7.821 | 0.094 |
| NUE | Drip | 1 | 10 | 42 | < .001 | 330 | < .001 | -2.159 | 0.031 |
|  | Furrow | 6 | 43 |  |  |  |  | -4.899 | <.001 |
|  | Sprinkling | 1 | 8 |  |  |  |  | -2.550 | 0.011 |

**Table S7** Detailed information about the 39 explanatory variables/features included for modelling *RR*_Y_, *RR*_WPc_, and *RR*_NUEf_ using Decision Tree-based algorithms. Calculated Evapotranspiration (CET) was estimated based on either directly reported ET values or derived values based on water productivity and yield data from each study.

| **Feature Name** | **Abbreviation** | **Mean (if continuous)** | **Range (if continuous)** | **Labels (if discrete)** |
| --- | --- | --- | --- | --- |
| Study Identification | Study ID | NA | NA | 1-143 |
| Region | Region | NA | NA | 1 (North), 2 (East), 3 (Center), 4 (Northwest) |
| Mean Annual Precipitation | MAP | 541.2 | 683.2 | NA |
| Mean Annual Temperature | MAT | 11.9 | 10.9 | NA |
| Soil Texture | Soil Texture | NA | NA | 1 (Loam), 2 (Silty loam), 3(Clay loam), 4 (Sandy loam), 5 (Sandy) |
| Wheat Type | Wheat type | NA | MA | 1 (Winter wheat) 2 (Spring wheat) |
| Highest Growing Season Temperature | HGT | 36.1 | 13.0 | NA |
| Lowest Growing Season Temperature | LGT | -11.7 | 24.2 | NA |
| Mean Growing Season Temperature | MGT | 12.4 | 19 | NA |
| Climate Conditions | Climate | NA | NA | 1 (Humid), 2 (Semi-humid), 3(Semi-arid), 4 (Arid) |
| Initial Soil Organic Carbon | SOC | 9.3 | 16.2 | NA |
| Initial Soil Total N | Total N | 0.95 | 1.5 | NA |
| Initial Soil Available N | AN | 68.7 | 139.1 | NA |
| Initial Soil Available P | AP | 23.9 | 64.6 | NA |
| Initial Soil Available K | AK | 143.7 | 320.8 | NA |
| Irrigation Method | Irrigation | NA | NA | 1 (Sprinkling), 2 (Furrow), 3 (Drip) |
| Cumulative Growing-season Precipitation | CGP | 167.5 | 359.0 | NA |
| Before Sowing Irrigation | BSI | 13.6 | 181.0 | NA |
| Over Winter Irrigation | OWI | 7.2 | 120.0 | NA |
| Reviving-stage (Feekes-3) Irrigation | RI | 2.5 | 91.3 | NA |
| Erection-stage (Feekes-4) Irrigation | EI | 2.5 | 164.9 | NA |
| Jointing-stage (Feekes-6) Irrigation | JI | 35.9 | 280.0 | NA |
| Booting-stage (Feekes-10) Irrigation | BI | 5.9 | 120.0 | NA |
| Heading-stage (Feekes-10.3) Irrigation | HI | 4.7 | 105.0 | NA |
| Anthesis-stage (Feekes-10.5.2) Irrigation | AI | 16.8 | 120.0 | NA |
| Filling-stage (Feekes-10.5.4) Irrigation | FI | 12.1 | 165.0 | NA |
| Milking-stage (Feekes-11.1) Irrigation | MI | 3.6 | 187.5 | NA |
| Cumulative Growing-season Irrigation | CGI | 126.0 | 705.1 | NA |
| Cumulative Growing-season Water Input (Precipitation + Irrigation) | CGWI | 293.5 | 660.3 | NA |
| Calculated Evapotranspiration | CET | 415.0 | 1157.9 | NA |
| Before Sowing N Input | BSNI | 104.6 | 450.0 | NA |
| Tillering-stage (Feekes-2) N Input | TNI | 1.4 | 142.5 | NA |
| Reviving-stage (Feekes-3) N Input | RNI | 10.1 | 345.0 | NA |
| Jointing-stage (Feekes-6) N Input | JNI | 55.9 | 337.5 | NA |
| Booting-stage (Feekes-10) N Input | BNI | 1.2 | 75.0 | NA |
| Heading-stage (Feekes-10.3) N Input | HNI | 1.9 | 111.0 | NA |
| Anthesis-stage (Feekes-10.5.2) N Input | ANI | 3.7 | 180.0 | NA |
| Filling-stage (Feekes-10.5.4) N Input | FINI | 2.2 | 90.0 | NA |
| Cumulative Growing-season N Input | CGNI | 187.7 | 525.0 | NA |

**Table S8.** Meta-regression for the response ratio of grain yield (*RR*_Y_), water use efficiency (*RR*_WUE_), and nitrogen use efficiency (*RR*_NUE_) of wheat against to concentration (*x*) of soil initial available nitrogen (AN) and initial available potassium (AK). n shorts for the number of observations.

| Property | Concentration | Equation | *p* | *R*^2^ | n |
| --- | --- | --- | --- | --- | --- |
| *RR*_Y_ | AN | *y* = - 0.006 *x* + 0.766 | <.0001 | 0.26 | 594 |
|  | AK | *y* = 0.001 *x* + 0.192 | <.0001 | 0.06 | 747 |
| *RR*_WUE_ | AN | *y* = - 0.005 *x* + 0.521 | <.0001 | 0.26 | 269 |
|  | AK | *y* = 0.001 *x* + 0.015 | <.0001 | 0.03 | 362 |
| *RR*_NUE_ | AN | *y* = 0.002 *x* - 0.207 | 0.006 | 0.11 | 71 |
|  | AK | *y* = 0.001 *x* - 0.115 | 0.03 | 0.06 | 79 |

**
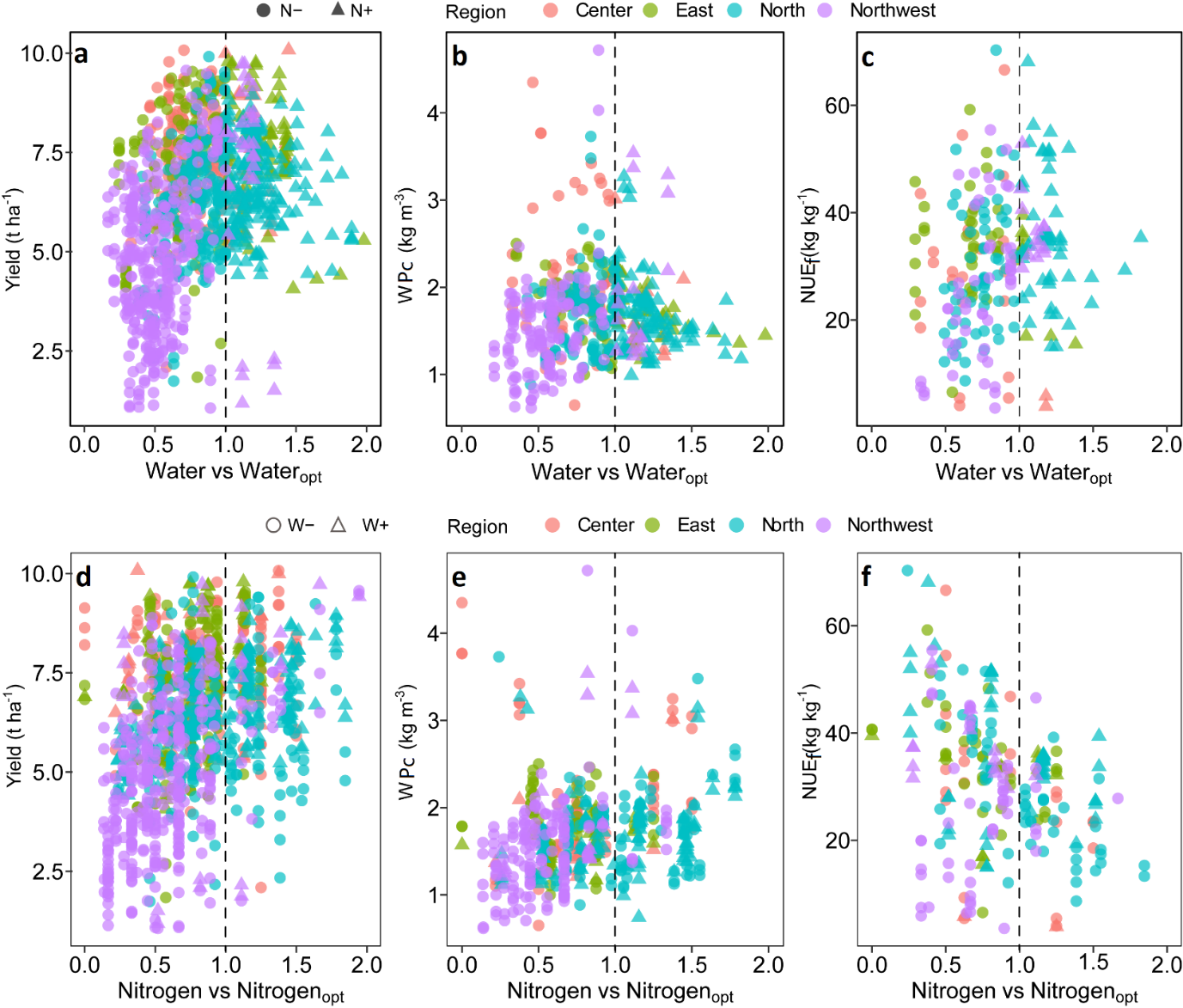
**

**Fig. S1.** Scatter plots show the effect of the ratio of total water input to the optimal water input on wheat a) grain yield, b) water productivity (WP_c_), c) fertiliser nitrogen use efficiency (NUE_f_) and the ratio of total N input to the optimal nitrogen input on d) grain yield, e) WP_c_, and f) NUE_f_. The optimal water and nitrogen inputs depend on regions (check Table 2−2). The grey line indicates that the total water or nitrogen input equal to optimal water or nitrogen input. Note the different scales among the graphs.

**
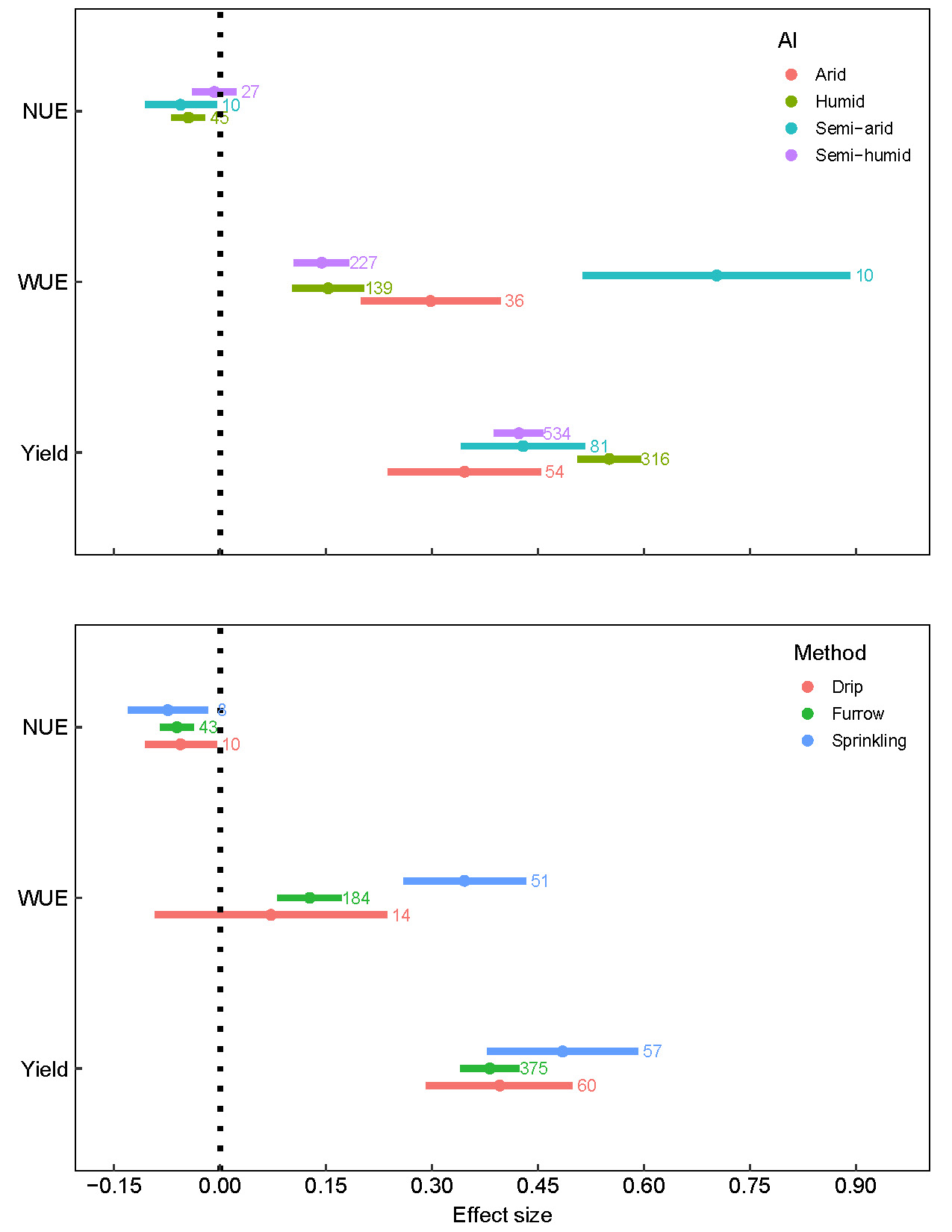
**

**Fig. S2.** The effect size of climate conditions (upper panel) and irrigation methods (lower panel) on grain yield, water use efficiency (WUE), and nitrogen use efficiency (NUE) of wheat. Effect size stands for the weighted effect size between treatment and control. Error bars represent the 95% confidence intervals. The sample size of each variable was displayed adjacent to each bar. AI shorts for aridity index.

**
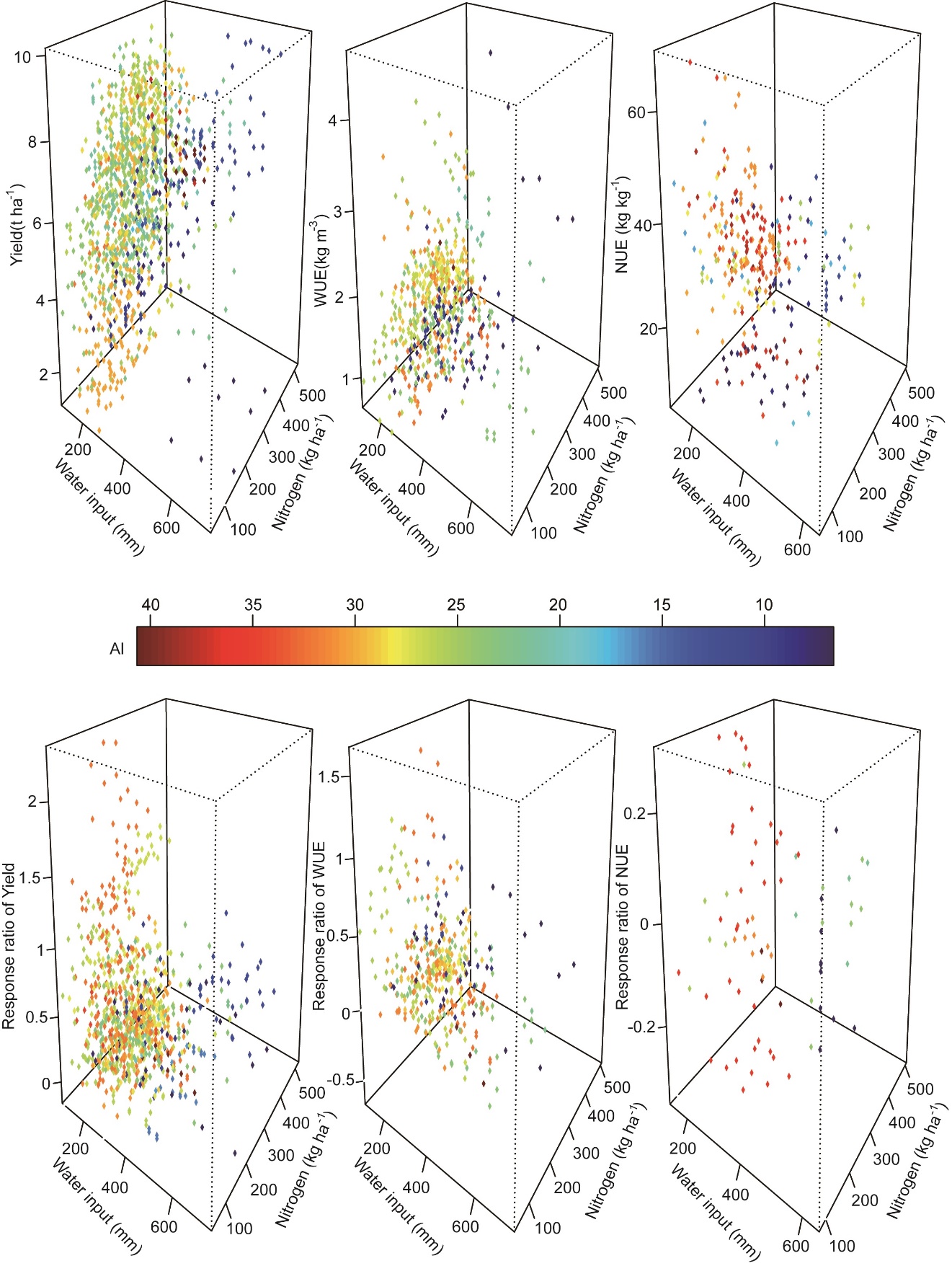
**

**Fig. S3.** 3D plot shows grain yield, water use efficiency (WUE), nitrogen use efficiency (NUE), and the response ratio of grain yield (*RR*_Y_), water use efficiency (*RR*_WUE_), and nitrogen use efficiency (*RR*_NUE_) of wheat in relation with total water and nitrogen input. AI indicates aridity index, the AI values of 0-10, 10-20, 20-30, and >30 correspond to arid or semi-arid, semi-humid, and humid environments, respectively.

**List of references used for the meta-analysis**

Cai, R.G., D. Zhang, M. Zhang, R.Q. Li and W.P. Wang. 2014. Effect of nitrogen application rate on dry matter accumulation and grain yield of winter wheat under irrigated and rainfed conditions. Journal of Triticeae Crops 34: 194-202. doi:10. 7606/j. issn, 1009-1041. 2014. 02. 09.

Cao, C.Y., H.K. Dang, C.L. Zhang, L. Guo, J.Y. Ma and K.J. Li. 2016. Effects of different irrigation regime on yield, water consumption and water use efficiency of winter wheat. Acta Agriculture Boreali-Sinica 31: 17-24. doi:10. 7668/hbnxb. 2016. S1. 003.

Chen, J., Z.M. Ma, X.D. Lv and T.T. Liu. 2016. Influence of different levels of irrigation and nitrogen application on the root growth and yield of spring wheat under permanent raised bed. Chinese Journal of Applied Ecology 27: 1511-1520. doi:10. 13287/j. 1001-9332. 201605. 033.

Chen, J., Y.C. Wang, H. Li, L.G. Wang, J.J. Qiu and B.L. Xiao. 2015. Characteristics soil nitrate nitrogen distribution, accumulation and nitrogen balance in winter wheat field under drip fertigation. Journal of Plant Nutrition and Fertilizer 21: 927-935. doi:10. 11674/zwyf. 2015. 0411.

Chen, K.L., J.H. Zhao, Y.J. Ma and J. Xu. 2017. Effects of different water and nitrogen treatments on the growth, yield, water and nitrogen utilization of spring wheat under drip irrigatiion in Altay area. Journal of Xinjiang Agricultural University 40: 85-91.

Cheng, M.Z., L. Li, C. Ma, X.T. Song, Z.Y. Lu, Q. Sun, et al. 2014. Effect of water and nitrogen coupling on high yield and high efficiency of water and fertilizer of winter wheat Journal of Triticeae Crops 34: 380-387. doi:10. 7606/j. issn. 1009-1041. 2014. 03.14.

Cui, Y., H.Z. Zhang, Q. Zhao and J.H. Liu. 2018. Effect of irrigation and fertilization on dry matter accumulation and yield control of drip irrigation winter wheat. Xinjiang Agricultural Sciences 55: 618-626. doi:10. 6048/j. issn. 1001-4330. 2018. 04. 004.

Dai, J., Z.L. Wang, M.H. Li, G. He, Q. Li, H.B. Cao, et al. 2016. Winter wheat grain yield and summer nitrate leaching: Long-term effects of nitrogen and phosphorus rates on the Loess Plateau of China. Field Crops Research 196: 180-190. doi:http://dx.doi.org/10.1016/j.fcr.2016.06.020.

Dai, Z.M., Y. Li, H. Zhang, L.Y. Wang, X.L. Zhang, Y. Li, et al. 2015. Effects of different irrigation treatments on nitrogen accumulation and translocation after anthesis in wheat. Journal of Triticeae Crops 35: 1712-1718. doi:10. 7606/j. issn. 1009-1041. 2015.12.15.

Dang, J.Y., X.X. Pei, D.Y. Zhang, J. Zhang, J.A. Wang and M.F. Cheng. 2019. Regulation effects of irrigation methods and nitrogen application on soil water and nitrate nitrogen and wheat growth and development. Chinese Journal of Applied Ecology 30: 1161-1169. doi:10.13287/j.1001-9332.201904.034

Dang, T.H., G.X. Cai, S.L. Guo, M.D. Hao and L.K. Heng. 2006. Effect of nitrogen management on yield and water use efficiency of rainfed wheat and maize in Northwest China. Pedosphere 16: 495-504. doi:https://doi.org/10.1016/S1002-0160(06)60080-5.

Dong, Z.Q., L.H. Zhang, L.H. Lv, Q. Li, S.B. Liang and X.L. Jia. 2015. Effects of different irrigation methods on photosynthetic rate and yield of winter wheat. Agricultural Research in the Arid Areas 33: 1-7. doi:10. 7606/j. issn. 1000-7601. 2015. 06. 01.

Du, J.J. and F.C. Li. 2017. Experimental study on the growth and yield of winter wheat under different irrigations and nitrogen applications. Journal of Irrigation and Drainage 36: 30-34. doi:10.13522/j.cnki.ggps.2017.09.006.

Du, J.J., S.X. Li, Y.J. Gao, S.Q. Li, C.H. Wang and X.H. Tian. 1999. Effects of nitrogen fertilizer on the mechanism of adaptation to water stress and water use of winter wheat. Acta University Agriculturae Boreali-Occidentalis 27: 1-5.

Duan, W.X., Z.W. Yu, Y.L. Zhang, D. Wang and Y. Shi. 2012. Effects of nitrogen fertilizer application rate on nitrogen absorption, translocation and nitrate nitrogen content in soil of dryland wheat Scientia Agricultura Sinica 45: 3040-3048. doi:10.3864/j.issn.0578-1752.2012.15.004

Duan, W.X., Z.W. Yu, Y.L. Zhang, D. Wang, Y. Shi and Z.Z. Xu. 2014. Effects of nitrogen application on biomass accumulation, remobilization, and soil water contents in a rainfed wheat field. Turkish Journal of Field Crops 19: 25-34. doi:https://doi.org/10.17557/tjfc.45522.

Duan, W.X., Y.L. Zhang, Y. Shi and Z.W. Yu. 2011. Effects of soil-moisture monitoring supplemental irrigation on water consumption characteristics and nitrogen accumulation and translocation in different wheat cultivars. Plant Nutrition and Fertilizer Science 17: 1309-1317.

Fang, Q., L. Ma, Q. Yu, L.R. Ahuja, R.W. Malone and G. Hoogenboom. 2010. Irrigation strategies to improve the water use efficiency of wheat-maize double cropping systems in North China Plain. Agricultural Water Management 97: 1165-1174. doi:https://doi.org/10.1016/j.agwat.2009.02.012.

Feng, B., L.A. Kong, B. Zhang, J.S. Si, S.D. Li and F.H. Wang. 2012. Effect of nitrogen application level on nitrogen use efficiency in wheat and soil nitrate-N content under bed planting condition Acta Agronomica Sinica 38: 1107-1114. doi:10.3724/SP.J.1006.2012.01107

Feng, S.W., S.B. Gu, H.B. Zhang and D. Wang. 2017. Root vertical distribution is important to improve water use efficiency and grain yield of wheat. Field Crops Research 214: 131-141. doi:https://doi.org/10.1016/j.fcr.2017.08.007.

Feng, W.S., W.Z. Tian, S.H. Wu, S.L. Zhang, H.X. Wen, X.P. Zhang, et al. 2017. Effects of irrigation on yield and water use efficiency of dryland wheat in Yuxi dryland wheat area. Crop Research 31: 228-231. doi:10.16848 /j. cnki. issn. 1001-5280. 2017. 03. 04.

Guo, S., H. Zhu, T. Dang, J. Wu, W. Liu, M. Hao, et al. 2012. Winter wheat grain yield associated with precipitation distribution under long-term nitrogen fertilization in the semiarid Loess Plateau in China. Geoderma 189: 442-450. doi:10.1016/j.geoderma.2012.06.012.

Guo, Z.J., Y.L. Zhang, J.Y. Zhao, Y. Shi and Z.W. Yu. 2014. Nitrogen use by winter wheat and changes in soil nitrate nitrogen levels with supplemental irrigation based on measurement of moisture content in various soil layers. Field Crops Research 164: 117-125. doi:http://dx.doi.org/10.1016/j.fcr.2014.05.016.

Han, M.K., M. Zhang, J.P. Li, X.X. Xu, Y.Q. Wang, Y.H. Zhang, et al. 2017. Influence of different soil water storage before sowing on yield and water use efficiency of winter wheat in the Heilonggang Plain. Journal of Triticeae Crops 37: 1201-1208.

Han, Y.Y., G.Y. Wang, X.B. Zhou, Y.H. Chen and P. Liu. 2014. Radiation use efficiency and yield response of winter wheat to planting patterns and irrigation in northern China. Agronomy Journal 106: 168-174. doi:10.2134/agronj2013.0252.

Hao, X.C., J.B. Li, S.A. Zhang, J. Guo, S.Y. Chen, Z.L. Shi, et al. 2017. Effects of different irrigation condition on yield and water use efficiency of winter wheat Journal of Hebei Agricultural Sciences 21: 1-6. doi:10. 16318/j. cnki. hbnykx. 2017. 03. 001.

Hu, Y.Y., W.L. Wan, J.L. Wang and M. Diao. 2018. Effects of different water and nitrogen application rates on the accumulation and translocation of nitrogen and yield of spring wheat under drip irrigation. Journal of Shihezi University (Natural Science) 36: 448-456. doi:10. 13880/j. cnki. 65-1174/n. 2018.04. 009.

Huang, L., W.P. Yang, X.Q. Hu, Y. Tao, S.M. Yao and X.Q. Ou. 2016. Effects of irrigation and nitrogen interaction on water consumption characteristics and nitrogen utilization of winter wheat. Journal of Soil and Water Conservation 30: 168-174. doi:10. 13870/j. cnki. stbcxb. 2016. 02. 030.

Huang, L.F., Y.G. Liu, Q. Lin and J.T. Li. 2019. Effect of supplemental irrigation on nitrogen translation and high-yield wheat yield in drylands Chinese Journal of Eco-Agriculture 17: 905-908. doi:10. 3724/SP.J.1011.2009.00905

Ji, Y.Z., X.T. Ju, X.Y. Liu, L.J. Zhang, X. Li and N. Liu. 2010. Ampact of different Nitrogen Application on Nitrogen Movement and Gaseous Loss of Winter Wheat Fields. Journal of Soil and Water Conservation 24: 113-118. doi:10.13870/j.cnki.stbcxb.2010.03.043.

Jiang, D.Y. and Z.W. Yu. 2008. Effcets of irrigation rate on the yield of wheat and the content of soil nitrate. Chinese Journal of Soil Science 39: 703-705.

Jiang, L.N., J.L. Ma, B.T. Fang, J.H. Ma, C.X. Li, Z.M. Wang, et al. 2019. Effect of lower water and nitrogen supply on grain yield and dry matter remobilization of organs in different layers of winter wheat plant in northern Henan province Acta Agronomica Sinica 45: 957-966. doi:10.3724/SP.J.1006.2019.81068

Jiang, X.G., Z.W. Yu, Y.Y. Zhang and Y. Shi. 2018. Effects of different fertilization and irrigation methods on flag leaf chlorophyll fluorescence characteristics and yield of wheat. Shandong Agricultural Sciences 50: 71-75. doi:10.14083/ j. issn. 1001- 4942. 2018. 01. 014.

Jiao, Y.P., X.Q. Meng, X.L. Ma and R.M. Feng. 2012. Effects of nitrogen application on the yields of wheat and maize distribution of soil nutrients. South-to North Water Diversion and Water Science & Technology 10: 103-108. doi:10. 3724/SP. J. 1201. 2012. 03103.

Jin, X.K. and T.K. Zhao. 2017. Effects of supplemental irrigation based on soil moisture content measuring and nitrogen fertilizer application on winter wheat yield, nitrogen absorption and distribution. Journal of Soil and Water Conservation 31: 233-239. doi:10. 13870/j. cnki. stbcxb.2017. 02. 039.

Jin, X.X., X.Y. Zhang, S.Y. Chen, H.Y. Sun, Y.M. Wang, L.W. Shao, et al. 2009. Effect of different irrigation frequency and amount on nitrogen uptake,translocation of winter wheat. Acta Agriculture Boreali-Sinica 24: 112-118.

Kong, D., Y. Yan, Y. Duan, W.H. Lu and H.Y. Xu. 2008. Field experiment study on growth and yields of winter wheat under different water and nitrogen treatments. Transactions of the CSAE 24: 36-40.

Li, C.J., X.X. Wen, X.J. Wan, Y. Liu, J. Han, Y.C. Liao, et al. 2016. Towards the highly effective use of precipitation by ridge-furrow with plastic film mulching instead of relying on irrigation resources in a dry semi-humid area. Field Crops Research 188: 32-73. doi:http://dx.doi.org/10.1016/j.fcr.2016.01.013.

Li, H.R., B. Jia, H.G. Wang, D.X. Li and R.Q. Li. 2017. Effects of irrigation and nitrogen treatments on dry matter accumulation and yield of a strong gluten winter wheat Journal of Hebei Agricultural Sciences 21: 10-17. doi:10. 16318/j. cnki. hbnykx. 2017. 05. 003.

Li, J.M., S. Inanaga, Z.H. Li and A.E. Eneji. 2005. Optimizing irrigation scheduling for winter wheat in the North China Plain. Agricultural Water Management 76: 8-23. doi:https://doi.org/10.1016/j.agwat.2005.01.006.

Li, J.P., Z.M. Wang, Q. Zhang, X.X. Xu, Y.Q. Wang, Y. Liu, et al. 2016. Effect of micro-sprinkling irrigation and nitrogen application rate on grain filling and nitrogen uptake and utilization in winter wheat. Acta Agriculturae Boreali-Occidentalis Sinica 31: 1-10. doi:10. 7668/hbnxb. 2016. S1. 001.

Li, L., H.T. Wang, Y.H. Xie and L. Zhang. 2013. Effects of watering and nitrogen fertilizaiton on the growth, grain yield, and water and nitrogen use efficiency of winter wheat. Chinese Journal of Applied Ecology 24: 1367-1373. doi:10.13287/j.1001-9332.2013.0301.

Li, N.N., Y. Liu, Y.X. Xie, Y.J. Zhu, C.Y. Wang and T.C. Guo. 2013. Effects of water and nitrogen interaction on starch content and yield of winter wheat. Journal of Triticeae Crops 33: 103-107.

Li, Q.Q., C.Y. Bian, X.H. Liu, C.J. Ma and Q.R. Liu. 2015. Winter wheat grain yield and water use efficiency in wide-precision planting pattern under deficit irrigation in North China Plain. Agricultural Water Management 153: 71-76. doi:http://dx.doi.org/10.1016/j.agwat.2015.02.004.

Li, Q.Q., Y.H. Chen, M.Y. Liu, X.B. Zhou, S.L. Yu and B.D. Dong. 2008. Effects of irrigation and planting patterns on radiation use efficiency and yield of winter wheat in North China. Agricultural Water Management 95: 469-476. doi:https://doi.org/10.1016/j.agwat.2007.11.010.

Li, Q.Q., Y.H. Chen, L. Mengyu, X.B. Zhou, S.L. Yu and B.D. Dong. 2008. Effects of irrigation and straw mulching on microclimate characteristics and water use efficiency of winter wheat in north China. Plant Production Science 11: 161-170. doi:https://doi.org/10.1626/pps.11.161.

Li, T.L., Y.H. Xie, J.P. Hong, Q. Feng, C.H. Sun and Z.W. Wang. 2013. Effects of nitrogen application rate on photosynthetic characteristics, yield, and nitrogen utilization in rainfed winter wheat in southern Shanxi province. Acta Agronomica Sinica 39: 704-711. doi:10.3724/SP.J.1006.2013.00704

Li, X.L., H.R. Li, W.P. Hao, W.Y. Zhang and C.H. Wang. 2018. Impact of drip fertigation on yields and water use efficiency of wheat-maize rotation in North China. Journal of Irrigation and Drainage 37: 18-28. doi:10.13522/j.cnki.ggps.2017.0687.

Li, Y., H.J. Liu and G.H. Huang. 2016. The effect of nitrogen rates on yields and nitrogen use efficiencies during four years of wheat-maize rotation cropping seasons. Agronomy Journal 108: 2076-2088. doi:10.2134/agronj2015.0610.

Li, Y., R.B. Zhang, H.J. Liu and G.H. Huang. 2013. Effect of application rate of nitrogen on nitrate distribution and accumulation in soil profile and their effects on crop yield in winter wheat-summer maize rotation system. Journal of Beijing Normal University (Natural Science) 49: 214-220.

Li, Y.B., L.M. Zheng, S.H. Liao, H. Zhu and H.J. Liu. 2005. Effects of different patterns of irrigation and N application on grain yield and utilization ratio of water and nutrient of winter wheat in Beijing suburb. Journal of Triticeae Crops 25: 51-56.

Li, Y.M., L.Y. Zhang and Z.G. Li. 1996. A study on the practice of spring watering and nitrogen-dressing for improving grain yield and quality of winter wheat. Journal of Hebei Agricultural University 19: 1-6.

Lin, X., D. Wang, S.B. Gu, P.J. White, K. Han, J. Zhou, et al. 2016. Effect of supplemental irrigation on the relationships between leaf ABA concentrations, tiller development and photosynthate accumulation and remobilization in winter wheat. Plant Growth Regulation 79: 331-343. doi:10.1007/s10725-015-0137-8.

Liu, C., K. Wang and X. Zhang. 2012. Responses of N_2_O and CH_4_ fluxes to fertilizer nitrogen addition rates in an irrigated wheat-maize cropping system in northern China. Biogeosciences 9: 839-850. doi:10.5194/bg-9-839-2012.

Liu, C., G.Z. Zhang, H.J. Zhu and Y.J. Zhu. 2015. Effects of irrigation and nitrogen-sulfur combined application on grain yield and water use efficiency in winter wheat. Journal of Henan Agricultural Sciences 44: 13-18. doi:10. 15933/ j. cnki. 1004-3268. 2015. 01. 004.

Liu, L.P., Z. Ouyang, L.F. Wu and Z.Z. Sun. 2011. Effect of irrigation schedules on population quality and grain yield of winter wheat under different densities. Journal of Triticeae Crops 31: 1116-1122.

Liu, X.G., F.C. Zhang, Y.F. Tian and Z.J. Li. 2009. Interactive impact of water and nitrogen on group yield of spring wheat and use of water and nitrogen in Shiyang River Basin. Journal of Northwest A&F University (Natural Science) 37: 107-113. doi:10.13207/j.cnki.jnwafu.2009.03.019.

Liu, X.J., X.T. Ju, F.S. Zhang, J.R. Pan and P. Christie. 2003. Nitrogen dynamics and budgets in a winter wheat-maize cropping system in the North China Plain. Field Crops Research 83: 111-124. doi:https://doi.org/10.1016/S0378-4290(03)00068-6.

Liu, Z.J., Z.J. Chen, P.Y. Ma, Y. Meng and J.B. Zhou. 2017. Effects of tillage, mulching and N management on yield, water productivity, N uptake and residual soil nitrate in a long-term wheat-summer maize cropping system. Field Crops Research 213: 154-164. doi:http://dx.doi.org/10.1016/j.fcr.2017.08.006.

Ma, G., P.P. Zhang, C.Y. Wang, W.X. LIu, M.W. Zhang, D.Y. Ma, et al. 2015. Regulation effect of irrigation and nitrogen on post-anthesis nitrogen accumulation, translocation and grain yield of high-yield wheat. Journal of Triticeae Crops 35: 798-805. doi:10. 7606/j. issn. 1009-1041. 2015. 06.10.

Ma, J.L., B.T. Fang, Y.W. Qiao, C.X. Li, Z.M. Wang, B.Z. Hao, et al. 2019. Effect of lower nitrogen application on canopy structure and photosynthesis of winter wheat grown under limited irrigation in northern Henan province. Journal of Triticeae Crops 39: 346-355. doi:10.7606/j. issn. 1009-1041. 2019. 03. 13.

Ma, X.H., D. Wang, Z.W. Yu, X.Z. Wang and Z.Z. Xu. 2010. Effect of irrigation regimes on water consumption characteristics and nitrogen distribution in wheat at different nitrogen applications. Acta Ecologica Sinica 30: 1955-1965.

Ma, Z.M., J. Chen, T.T. Liu and X.D. Lv. 2017. Effects of water and nitrogen coupling on root length density and yield of spring wheat in permanent raised-bed cropping system. Acta Agronomica Sinica 43: 1705-1714. doi:10.3724/SP.J.1006.2017.01705

Man, J.G., Y. Shi, Z.W. Yu and Y.L. Zhang. 2016. Root growth, soil water variation, and grain yield response of winter wheat to supplemental irrigation. Plant Production Science 19: 193-205. doi:https://doi.org/10.1080/1343943X.2015.1128097.

Man, J.G., Z.W. Yu, Y.L. Zhang, Y. Shi and L.Q. Wang. 2016. Water and nitrogen use of winter wheat under different supplemental irrigation regimes. Crop Science 56: 3237-3249. doi:10.2135/cropsci2015.08.0521.

Man, J.G., Z.W. Zhu and Y. Shi. 2017. Radiation interception, chlorophyll fluorescence and senescence of flag leaves in winter wheat under supplemental irrigation. Scientific Reports 7: 1-12. doi:https://xs.scihub.ltd/https://doi.org/10.1038/s41598-017-07414-2.

Men, H.W., Q. Zhang, X.L. Dai, Q. Cao, C.Y. Wang, X.H. Zhou, et al. 2011. Effects of different irrigation modes on winter wheat grain yield and water-and nitrogen use efficiency. Chinese Journal of Applied Ecology 22: 2517-2523. doi:10.13287/j.1001-9332.2011.0357.

Meng, W.W., Z.W. Yu, Y.L. Zhang, Y. Shi and D. Wang. 2015. Effects of supplemental irrigation on water consumption characteristics and grain yield in different wheat cultivars. Chilean Journal of Agricultural Research 75: 216-233. doi:10.4067/S0718-58392015000200011.

Meng, X.Y., Z.H. Wang, F.C. Li, K.Y. Li, C. Xue and S.X. Li. 2012. Effects of soil moisture before sowing and nitrogen fertilization on winter wheat yield and water use on Weibei Plain of Loess Plateau．. Chinese Journal of Applied Ecology 23: 369-375. doi:10.13287/j.1001-9332.2012.0044.

Ning, G.F., Z.J. Li, W.Y. Sun, W.P. Ma, S.W. Huang and B.Q. Zhao. 2010. Effects of nitrogen application on grain yield, nitrogen utilization and balance of winter wheat under limited irrigation condition. Plant Nutrition and Fertilizer Science 16: 1312-1318.

Qin, S.S., Z.J. Hou, Z.D. Wu, D.H. Ma and P. Huang. 2007. Effects of water and nitrogen coupling on nitrogen absorption and yield of winter wheat. Journal of drainage and irrigation machinery engineering 35: 440-447. doi:10. 3969/j. issn. 1674-8530. 16. 0100.

Ran, H., G.Y. Jiang, H.J. Xu, M.G. Li and M.C. Hao. 2015. Effect of irrigation frequency and nitrogen application rate on dry matter accumulatiion and yield of drip-irrigated spring wheat. Journal of Triticeae Crops 35: 379-386. doi:10. 7606/j. issn. 1009-1041. 2015. 03. 14.

Shi, W., Y.A. Tong, Y. Zhao and H.Z. Fan. 2006. Effect of nitrogen fertilizer and irrigation on apparent budget of soil nitrogen in winter wheat in Anthrosol. Journal of Triticeae Crops 26: 93-97.

Shi, Y., Z.W. Yu, J.N. He and Y.L. Zhang. 2016. Effects of supplemental irrigation by monitoring soil moisture on the water-nitrogen utilization of wheat and soil NO_3_^-^-N leaching. Chinese Journal of Applied Ecology 27: 445-452. doi:10. 13287/ j.1001-9332. 201602. 006.

Sui, J., J.D. Wang, S.H. Gong, Y.Q. Zhang, J.J. Cen and J. Guo. 2016. Coupling effects of water and nitrogen on water and nitrogen use efficiency and yield of winter wheat under drip irrigation. Journal of Drainage and Irrigation Machinery Engineering 34: 532-538. doi:10. 3969/j. issn. 1674-8530. 15. 0172.

Sun, M., P.Y. Guo, Z.Q. Gao, P. Wang, J. Shi and G.Y. Miao. 2010. Protein accumulation in grains of wheat cultivars differing in drought tolerance and its regulation by nitrogen application amount under irrigated and dryland conditions. Acta Agronomica Sinica 36: 486-495. doi:10.3724/SP.J.1006.2010.00486

Sun, X.S., Y.G. Liu and Q. Lin. 2017. Effects of supplement irrigation on carbon metabolism at later developing stages and yield of high-yield wheat in dry land. Chinese Agricultural Science Bulletin 33: 7-12.

Wang, B., W. Liu, Q. Xue, T. Dang, C. Gao, J. Chen, et al. 2013. Soil water cycle and crop water use efficiency after long-term nitrogen fertilization in Loess Plateau. Plant, Soil and Environment 59: 1-7. doi:https://doi.org/10.17221/207/2012-PSE.

Wang, B., Y.H. Zhang, B.Z. Hao, X.X. Xu, Z.G. Zhao, Z.M. Wang, et al. 2016. Grain yield and water use efficiency in extremely-late sown winter wheat cultivars under two irrigation regimes in the North China Plain. Plos ONE 11: e0153695. doi:10.1371/journal.pone.0153695.

Wang, C.Y., W.X. Liu, Q.X. Li, D.Y. Ma, H.F. Lu and W. Feng. 2014. Effects of different irrigation and nitrogen regimes on root growth and its correlation with above-ground plant parts in high-yielding wheat under field conditions. Field Crops Research 165: 138-149. doi:http://dx.doi.org/10.1016/j.fcr.2014.04.011.

Wang, J., W.Z. Liu, T.H. Dang and U.M. Sainju. 2013. Nitrogen fertilization effect on soil water and wheat yield in the Chinese Loess Plateau. Agronomy Journal 105: 143-149. doi:10.2134/agronj2012.0067.

Wang, L.L., J.A. Palta, W. Chen, Y.L. Chen and X.P. Deng. 2018. Nitrogen fertilization improved water-use efficiency of winter wheat through increasing water use during vegetative rather than grain filling. Agricultural Water Management 197: 41-53. doi:https://doi.org/10.1016/j.agwat.2017.11.010.

Wang, M., S.Q. Zhang, B.T. Fang, Q. Zheng and Y.H. Zhang. 2007. Effect of nitrogen applications on grain yield and nitrogen use efficiency of winter wheat in limited water supply. Chinese Agricultural Science Bulletin 23: 349-353.

Wang, Q., Y.S. Sun, T.T. Wang, G.Y. Fan, E.H. Zhang, F.R. Li, et al. 2009. Effect of different irrigation and nitrogen supply levels on spring wheat growth charateristics, water consumption and grain yield on recently reclaimed sandy farmlands in Heihe River basin. Arid Land Geography 32: 240-248. doi:10. 13826/j. cnki. cn65-1103/x. 2009. 02. 012.

Wang, S.J., X.H. Tian, T. Liu, X.C. Lu, D.H. You and S. Li. 2014. Irrigation, straw, and nitrogen management benefits wheat yield and soil properties in a dryland agro-ecosystem. Agronomy Journal 106: 2193-2201. doi:10.2134/agronj14.0211.

Wang, S.S., P.T. Meng, H. Liu, S.J. Wang and Q. Yuan. 2015. Research on growth characteritics and water use efficiency of winter wheat under different irrigation modes. China Rural Water and Hydropower 9: 115-118.

Wang, S.X., X.H. Tian, M.J. Li, Y.J. Ni, J. Li, H.Y. Li, et al. 2014. Water and nitrogen management on micronutrient concentrations in winter wheat. Agronomy Journal 106: 1003-1010. doi:10.2134/agronj13.0354.

Wang, X., Y. Shi, Z.J. Guo, Y.L. Zhang and Z.W. Yu. 2015. Water use and soil nitrate nitrogen changes under supplemental irrigation with nitrogen application rate in wheat field. Field Crops Research 183: 117-125. doi:http://dx.doi.org/10.1016/j.fcr.2015.07.021.

Wang, X.C., X.H. Wang, S.P. Xiong, M.X. M, S.J. Ding, K.Y. Wu, et al. 2015. Differences in nitrogen efficiency and nitrogen metabolism of wheat varieties under different nitrogen levels Scientia Agricultura Sinica 48: 2569-2579. doi:0.3864/j.issn.0578-1752.2015.13.009

Wang, X.Y., M.R. He, Y.H. Liu, H.H. Zhang, F. Li, F.X. Hua, et al. 2008. Interactive effects of irrigation and nitrogen fertilizer on nitrogen fertilizer recovery and nitrate-N movement across soil profile in a winter wheat field. Acta Ecologica Sinica 28: 685-694.

Wang, X.Y., C.Y. Zheng, Z.W. Yu and Z.Z. Xu. 2009. Effects of water-nitrogen interaction on soil water utilization by wheat and fructan content in wheat stem. Chinese Journal of Applied Ecology 20: 1876-1882. doi:10.13287/j.1001-9332.2009.0290.

Wang, Y.Z., X.W. Liu, H.Y. Sun, X.Y. Zhang and L.R. Zhang. 2013. Effects of water and nitrogen on root/shoot ratio and water use efficiency of winter wheat Chinese Journal of Eco-Agriculture 21: 282-289. doi:10.3724/SP.J.1011.2013.00282

Wang, Y.Z., X.Y. Zhang, X.Y. Zhang, L.W. Shao, S.Y. Chen and X.W. Liu. 2016. Soil water regime affecting correlation of carbon isotope discrimination with yield and water-use efficiency of winter wheat. Crop Science 56: 760-772. doi:10.2135/cropsci2014.11.0793.

Wang, Z.Q., W.W. Liang, X.Z. Xu, Z.Y. Xin and T.B. Lin. 2015. Effects of nitrogen fertilizer on flag leaves nitrogen assimilation, water and nitrogen use efficiency of irrigation-limited winter wheat. Journal of Irrigation and Drainage 34: 12-16. doi:10. 13522/j. cnki. ggps. 2015. 08. 003.

Wei, G.F., Y.G. Liu, W. Jiang, H.S. Zhang, Q. Lin and C.X. Zhao. 2013. Effects of different drip irrigation system on photosynthesis characteristics and water use efficiency of winter wheat. Acta Agriculture Boreali-Sinica 28: 149-156.

Wen, H.X., W.Z. Tian, G.H. Duan, S.F. Peng, Y.F. Zhang, S.Z. Lv, et al. 2018. Effect of water and nitrogen coupling on yied, water and nitrogen use efficiency of winter wheat. Journal of Seed Industry Guide 4: 9-13.

Wu, G.L., L.Y. Guo, Z.Y. Cui, Y. Li, Y.P. Yin, Z.L. Wang, et al. 2012. Differential effects of nitrogen managements on nitrogen, dry matter accumulation and transportation in late-sowing winter wheat. Acta Ecologica Sinica 32: 5128-5137. doi:10. 5846/stxb201202270263.

Wu, J.Z., M. Huang, Y.J. Li, G.Z. Fu and W.Z. Tian. 2017. Effect of limited irrigation on grain yield, protein content and water-nitrogen use efficiency in wheat under extremely-late sowing with high density. Journal of Triticeae Crops 37: 1349-1357. doi:10. 7606/j. issn. 1009-1041. 2017. 10. 11.

Wu, L.F., F.C. Zhang, P. Zhang, Z.J. Li and H.M. Zhou. 2011. Effect of irrigation and nitrogen fertilizer on growth and yield of spring wheat in Hexi oasis of Gansu. Journal of Northwest A&F University (Natural Science) 39: 55-63. doi:10.13207/j.cnki.jnwafu.2011.07.025.

Wu, Y.C., S.L. Zhou and Z.M. Wang. 2008. Effect of nitrogen fertilizer applications on yield, water and nitrogen use efficiency under limited irrigation of winter wheat in North China Plain. Journal of Triticeae Crops 28: 1016-1020.

Xu, J.K., Y. Shi, Z.W. Yu and J.Y. Zhao. 2017. Irrigation methods affect wheat flag leaf senescence and chlorophyll fluorescence in the North China Plain. International Journal of Plant Production 11: 361-378. doi:10.22069/ijpp.2017.3545.

Xue, L.H., J.L. Zhao and S.R. Sun. 2018. Effects of water-nitrogen coupling on photosynthetic characteristics, yield and water and nitrogen use efficiency of winter wheat under drip irrigation. Chinese Agricultural Science Bulletin 34: 12-19.

Yang, K.J., F.X. Wang, D. Ma, N. Song and Y.J. Lu. 2013. Effect of different drip irrigation quotas on water consumption and yield of spring wheat in arid region of Northwest China. Water Saving Irrigation 12: 12-19.

Yang, X.L., Y.L. Lu, Y. Ding, X.F. Yin, S. Raza and Y.A. Tong. 2017. Optimising nitrogen fertilisation: A key to improving nitrogen-use efficiency and minimising nitrate leaching losses in an intensive wheat/maize rotation (2008-2014). Field Crops Research 206: 1-10. doi:http://dx.doi.org/10.1016/j.fcr.2017.02.016.

Yang, X.Y., Z.W. Yu and Z.Z. Xu. 2009. Effects of irrigation regimes on water consumption characteristics and nitrogen accumulation and allocation in wheat. Acta Ecologica Sinica 29: 846-853.

Yao, Y.Q., Y.H. Wang, J.J. Lv, J. Zhang, J.H. Li, C.H. Wang, et al. 2005. Effects of different irrigation regime and fertilization time on yield and benefit of winter wheat. Tillage and Cultivation 2. doi:10.13605/j.cnki.52-1065/s.2005.02.006.

Yi, L.H., L. Wang, M.N. Zhang, P.P. Mao, J.Y. Dang, H.J. Wu, et al. 2017. Effect of irrigation methods on growth and water use efficiency of wnter wheat. Journal of Irrigation and Drainage 36: 14-19. doi:10.13522/j.cnki.ggps.2017.10.003.

Yin, X.S., S.W. Wang, X.P. Deng, Y.L. Li, W.J. Yang and H.N. Sun. 2018. Effect of irrigation on population dynamics, yield and water consumption characteristics of winter wheat at elongation under different nitrogen application levels in dryland. Research of Soil and Water Conservation 25: 179-186. doi:10.13869/j.cnki.rswc.2018.05.025.

Yu, H.L., E.H. Zhang, Q. Wang, Q.L. Liu, C.W. Liu, T.T. Wang, et al. 2013. Effects of irrigation and N supply levels on soil organic carbon, total nitrogen and grain yield of spring wheat on no-tillage farmland with standing stubble. Acta Prataculturae Sinica 22: 227-233. doi:10. 11686/cyxb20130330.

Zhang, D.J., D.D. Yang, J.H. Ma, D.D. Wang, Y.Y. Zhang, X.N. GUo, et al. 2017. Effect of drip irrigation and nitrogen regulation on WUE and grain filling of winter wheat based on soil moisture measurement. Journal of Henan Normal University (Natural Science Edition) 45: 48-55. doi:10. 16366 /j. cnki. 1000-2367. 2017. 01. 008.

Zhang, M.W., H. Wang, Y. Yi, J.F. Ding, M. Zhu, C.Y. Li, et al. 2017. Effect of nitrogen levels and nitrogen ratios on lodging resistance and yield potential of winter wheat (*Triticum aestivum* L.). PLoS ONE 12: e0187543. doi:https://doi.org/10.1371/journal.pone.0187543

Zhang, N., N.P. Wu, X.X. Xu, P.J. Wu and X.F. Cheng. 2015. Effect of nitrogen levels on dry matter and yield of winter wheat under drip irrigation. Chinese Agricultural Science Bulletin 31: 21-26.

Zhang, P.P., G. Ma, C.Y. Wang, S.S. Li, Y.X. Xie, D.Y. Ma, et al. 2017. Effect of irrigation and nitrogen application on grain amino acid composition and protein quality in winter wheat. PloS ONE 12: e0178494. doi:https://doi.org/10.1371/journal. pone.0178494

Zhang, S.A., Z.L. Li, Q.C. Qin, J.X. He and R.Q. Li. 2007. The influence of irrigation system in spring to winter wheat yield and water use efficiency. Acta Agriculturae Boreali-Occidentalis Sinica 22: 164-168.

Zhang, W., L.H. Li and X. Lv. 2016. Effects of nitrogen fertilizer at different levels on spatial and temporal distribution of wheat roots, nitrogen use efficieny and yield in wheat under drip irrigation. Acta Agriculturae Boreali-Occidentalis Sinica 25: 195-202. doi:10. 7606/ J. issn. 1004-1389. 2016. 02.006.

Zhang, X., Y.Q. Luo, S.Q. Zhang, M. Wang, Q. Zheng, H.Y. Feng, et al. 2006. Effect of nitrogen application on grain yield of winter wheat and accumulationof NO_3_^-^-N under water-saving cultivation system. Journal of Soil and Water Conservation 20: 102-105. doi:0.13870 /j .cnki .stbcxb.2006.04.024.

Zhang, X., Y.Q. Luo, S.Q. Zhang, Y.C. Wu, M. Wang and Z.M. Wang. 2007. Effect of different fertilization way on yield and protein content of winter wheat under water-saving cultivation system. Agricultural Research in the Arid Areas 25: 45-49.

Zhang, X.Y., X.X. Li, K. Wang, Z.D. Ao and D.W. Wang. 2013. Effects of irrigation amount on root distribution, yield of winter wheat and soil NO_3_^-^-N content under different nitrogen application rates. Journal of Agricultural University of Hebei 36: 13-19. doi:10.13320/j.cnki.jauh.2013.03.003.

Zhang, X.Y., W.L. Qin, S.Y. Chen, L.W. Shao and H.Y. Sun. 2017. Responses of yield and WUE of winter wheat to water stress during the past three decades-A case study in the North China Plain. Agricultural Water Management 179: 47-54. doi:http://dx.doi.org/10.1016/j.agwat.2016.05.004.

Zhang, Y.H., Q. Zhang, X.X. Xu, J.P. Li, B. Wang and S.L. Zhou. 2016. Optimal irrigation frequency and nitrogen application rate improving yield formation and water utilization in winter wheat under micro-sprinkling condition. Transactions of the Chinese Society of Agricultural Engineering 32: 88-95. doi:10.11975/j.issn.1002-6819.2016.05.013

Zhang, Y.L. and Z.W. Yu. 2008. Effects of irrigation amount on nitrogen uptake, distribution, use, and grain yield and quality in wheat Acta Agronomica Sinica 34: 870-878. doi:10.3724/SP.J.1006.2008.00870.

Zhang, Y.Q., J.D. Wang, S.H. Gong and J. Sui. 2015. Photosynthetic response of yield enhancement by nitrogen fertilization in winter wheat fields with drip irrigation. Transactions of the Chinese Society of Agricultural Engineering 31: 170-177. doi:10.3969/j.issn.1002-6819.2015.06.023

Zhang, Y.Q., J.D. Wang, S.H. Gong, D. Xu and J. Sui. 2017. Nitrogen fertigation effect on photosynthesis, grain yield and water use efficiency of winter wheat. Agricultural Water Management 179: 277-287. doi:http://dx.doi.org/10.1016/j.agwat.2016.08.007.

Zhao, L.J., L.H. Xue, Q.K. Sun and J.X. Zhang. 2016. Effect of different irrigation and nitrogen application on water consumption characteritics and the water and nitrogen use efficiencies under drip irrigation in winter wheat. Journal of Triticeae Crops 36: 1050-1059. doi:10.7606/j. issn. 1009-1041. 2016. 08.11.

Zhao, Y., F.X. Wang, Q. Zhou, K.J. Yang and Y.L. Zhang. 2016. Effect of drip tape distance and irrigation amount on spring wheat yield and water use efficiency. Chinese Agricultural Science Bulletin 32: 194-199.

Zheng, C.Y., Z.W. Yu, Y.L. Zhang, D. Wang and Z.Z. Xu. 2010. Effects of irrigation amount on water use characteristics and grain yield of wheat under different nitrogen application rates. Chinese Journal of Applied Ecology 20: 2799-2805. doi:10.13287/j.1001-9332.2010.0414.

Zhong, Y.Q.W. and Z.P. Shangguan. 2014. Water consumption characteristics and water use efficiency of winter wheat under long-term nitrogen fertilization regimes in Northwest China. PloS ONE 9: e98850. doi:10.1371/journal.pone.0098850.
