# Supplementary material for: Optimizing Wheat Yield, Water, and Nitrogen Use Efficiency With Water and Nitrogen Inputs in China: A Synthesis and Life Cycle Assessment
Source: Front Plant Sci. 2022 Jun 16;13:930484. doi: 10.3389/fpls.2022.930484 (PMC9244784; doi:10.3389/fpls.2022.930484)

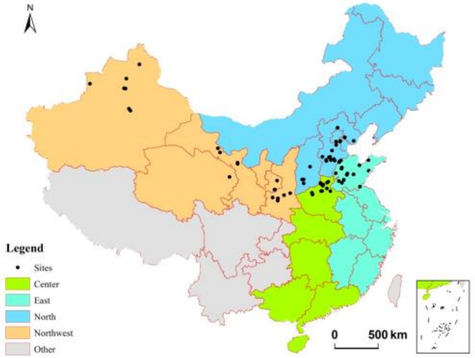

126 studies from peer-reviewed published journal articles from 1980 to 2018 were searched for using the ISI Web of Science and China's Knowledge Resource Integrated Database, including 1020 yield, 437 WP_c, and 82 NUE_f paired observations.

Meta−analysis

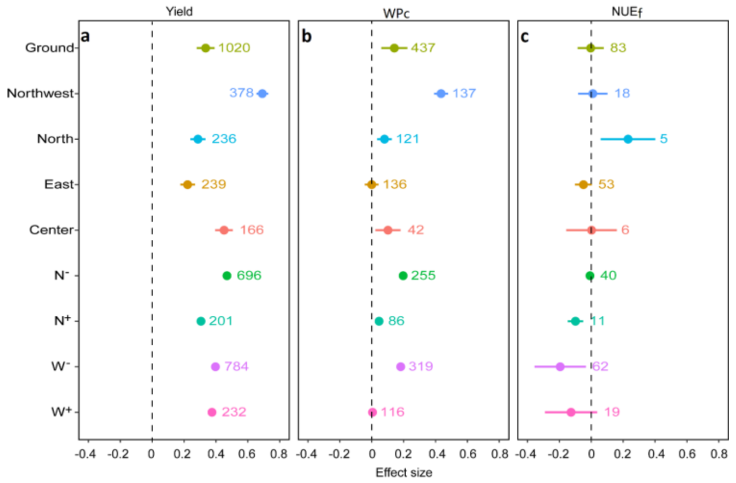

Structural Equation Modeling

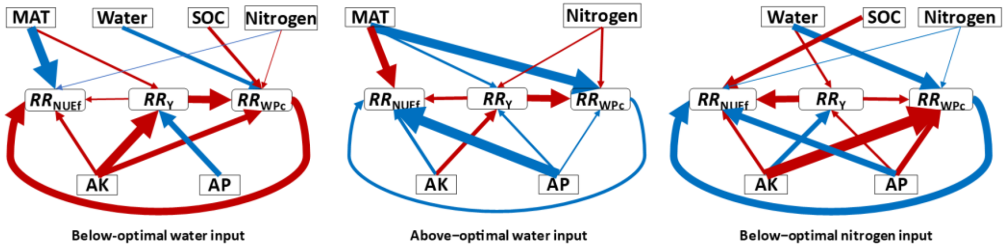

Decision tree-based modelling

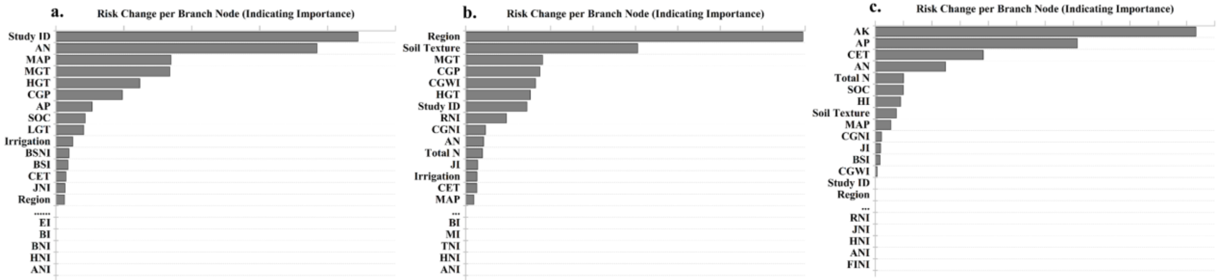

Life Cycle Assessment

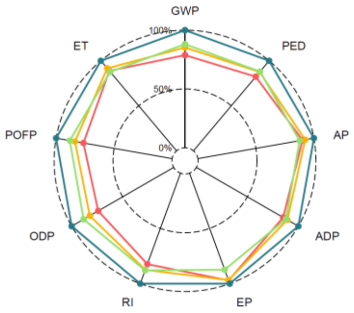

Supplement: Supplementary file 2 [file Image_1.PDF]
